# Tenogenic Induction From Induced Pluripotent Stem Cells Unveils the Trajectory Towards Tenocyte Differentiation

**DOI:** 10.3389/fcell.2022.780038

**Published:** 2022-03-09

**Authors:** Yuki Yoshimoto, Akiyoshi Uezumi, Madoka Ikemoto-Uezumi, Kaori Tanaka, Xinyi Yu, Tamaki Kurosawa, Shinsei Yambe, Kazumitsu Maehara, Yasuyuki Ohkawa, Yusuke Sotomaru, Chisa Shukunami

**Affiliations:** ^1^ Department of Molecular Biology and Biochemistry, Biomedical Sciences Major, Graduate School of Biomedical and Health Sciences, Hiroshima University, Hiroshima, Japan; ^2^ Muscle Aging and Regenerative Medicine, Tokyo Metropolitan Institute of Gerontology, Tokyo, Japan; ^3^ Division of Transcriptomics, Medical Institute of Bioregulation, Kyushu University, Fukuoka, Japan; ^4^ Laboratory of Veterinary Pharmacology, Department of Veterinary Medical Sciences, Graduate School of Agriculture and Life Sciences, Tokyo University, Tokyo, Japan; ^5^ Natural Science Center for Basic Research and Development, Hiroshima University, Hiroshima, Japan

**Keywords:** tendon, iPSCs, tenogenic differentiation, single cell analysis, Scx, retinoic acid, ligament

## Abstract

The musculoskeletal system is integrated by tendons that are characterized by the expression of scleraxis (Scx), a functionally important transcription factor. Here, we newly developed a tenocyte induction method using induced pluripotent stem cells established from *ScxGFP* transgenic mice by monitoring fluorescence, which reflects a dynamic differentiation process. Among several developmentally relevant factors, transforming growth factor-beta 2 (TGF-β2) was the most potent inducer for differentiation of tenomodulin-expressing mature tenocytes. Single-cell RNA sequencing (scRNA-seq) revealed 11 distinct clusters, including mature tenocyte population and tenogenic differentiation trajectory, which recapitulated the *in vivo* developmental process. Analysis of the scRNA-seq dataset highlighted the importance of retinoic acid (RA) as a regulatory pathway of tenogenic differentiation. RA signaling was shown to have inhibitory effects on entheseal chondrogenic differentiation as well as TGF-β2-dependent tenogenic/fibrochondrogenic differentiation. The collective findings provide a new opportunity for tendon research and further insight into the mechanistic understanding of the differentiation pathway to a tenogenic fate.

## Introduction

Tendons and ligaments are dense regular fibrous connective tissue that connect muscle to bone and bone to bone, respectively. They transmit the mechanical force generated by the muscle to the bone or stabilize the joint, and therefore act as important components in the musculoskeletal system. Tendons and ligaments have similarities in terms of developmental origin, structure, and histological and physiological properties. We mainly used the term tendon in this study because there were no molecular markers that clearly distinguished these tissues.

The major cell population of tendon is a specialized type of fibroblasts called tenocytes. They are derived from the subdomain of the sclerotome, including the syndetome in the trunk, the lateral plate mesoderm in limbs, or the cranial neural crest in the craniofacial region (Schweitzer et al., 2010). The basic helix-loop-helix (bHLH) transcription factor scleraxis (Scx) is expressed in all tenogenic cells during development and is considered a distinctive marker of tenogenic lineage cells from the early embryonic stages and throughout development (Schweitzer et al., 2010; Sugimoto et al., 2013a). Transgenic (Tg) reporter mouse lines harboring enhanced green fluorescent protein (EGFP) under the control of the regulatory region of the *Scx* locus showed GFP expression that faithfully recapitulated the endogenous *Scx* expression pattern (Pryce et al., 2007; Sugimoto et al., 2013b). Scx is important as a marker of tenogenic cells and as a functional regulator of tenocyte differentiation (Shukunami et al., 2006; Shukunami et al., 2018). Scx has important roles in tendon formation (Murchison et al., 2007); recruitment of tenocyte progenitors (Huang et al., 2019); and maturation of tendons, ligaments, and enthesis (Murchison et al., 2007; Yoshimoto et al., 2017). Scx acts as a transcriptional activator of *collagen type 1* alpha 1 (*Col1a1*) (Léjard et al., 2007) and *tenomodulin* (*Tnmd*), a mature marker for tenocytes (Shukunami et al., 2006; Shukunami et al., 2018). Therefore, Scx is one of the most powerful transcription factors that endows the characteristics of tenocytes. However, the molecular mechanism by which Scx regulates tenogenic differentiation is not fully understood.

The transforming growth factor-beta (TGF-β) signaling pathway is critical for tendon development. Mice harboring a double mutant for *Tgfb2* and *Tgfb3*, or conditional deletion of *Tgfbr2* in mesenchymal cells, showed loss of most tendons and ligaments (Pryce et al., 2009). Transcriptomic analysis of developing mouse limb tendon cells has also highlighted the importance of TGF-β signaling in tendon development (Havis et al., 2014). TGF-β signaling activation by supplementation with TGF-β ligands stimulates the expression of tendon-related genes, including *Scx*, in mouse limb explants or C3H10T1/2 mesenchymal stem cells (MSCs) (Havis et al., 2014). Growth differentiation factor-8 (GDF-8), GDF-5, and other members of the TGF-β superfamily are reportedly involved in tenogenic differentiation (Mendias et al., 2008; Tan et al., 2012; Mienaltowski et al., 2014). Studies on fibroblast growth factor (FGF) signaling in tendon development have reported contradictory results. Both positive and negative actions of FGF on tendon development were reported (Edom-Vovard et al., 2002; Brent et al., 2003; Havis et al., 2014).

Although the factors that regulate tenogenic differentiation have gradually become clear, the detailed molecular mechanism that controls the dynamic differentiation processes is less well understood compared to that of other mesenchymal cell types. One of the obstacles to tendon research is the lack of a sound and reproducible culture system for tenogenic differentiation. Tenocytes can be isolated from tendon tissue by outgrowth or enzyme digestion, but the expansion of isolated cells is sometimes required because of the hypocellularity of the tendon. Repeated passages of isolated tenocytes affect cellular properties, including decreased expression of mature marker genes, ultimately leading to dedifferentiation (Shimada et al., 2014; Shukunami et al., 2018; Jo et al., 2019). Tendon stem cells residing in tendon fascicles (Bi et al., 2007) and paratenon sheath (Harvey et al., 2019) have been reported. However, it is still difficult to isolate sufficient numbers of these cells for *in vitro* research. Although *in vitro* tenogenic differentiation from embryonic stem cells (ESCs) and induced pluripotent stem cells (iPSCs) has also been reported, these systems still have problems, including complicated procedures (Nakajima et al., 2018) and poor tendon marker gene expression (Chen et al., 2009; Chen et al., 2012; Komura et al., 2020). Recently, Kaji et al. reported a system with high induction efficiency using *ScxGFP* ESCs, but this procedure is still relatively complicated because it is based on embryoid body (EB) formation (Kaji et al., 2021).

To address this problem, we induced tenocytes from *ScxGFP-*Tg-*mouse-derived* iPSCs by stepwise differentiation. We optimized the culture conditions by monitoring GFP expression and known tenogenic markers to establish a simple and effective method that is capable of inducing the generation of mature tenocytes expressing high levels of tendon marker genes. Single-cell RNA sequencing (scRNA-seq) during tenogenic induction confirmed the emergence of a mature tenocyte population positive for Tnmd and revealed a tenogenic differentiation trajectory. Furthermore, scRNA-seq dataset shed light on retinoic acid signaling, which has the inhibitory effect on Scx^+^ cell differentiation. The data presented here will provide meaningful information for a better understanding of tendon and ligament biology.

## Materials and Methods

### Mice

ICR mice used for isolation of MEFs and severe combined immunodeficient (SCID) mice used for teratoma formation were purchased from Japan SLC (Shizuoka, Japan), Shimizu Laboratory Supplies (Kyoto, Japan), and CLEA Japan (Shizuoka, Japan). Prior publications have detailed the generation and establishment of *ScxGFP* Tg mice (Sugimoto et al., 2013b). The mice were housed in a temperature-controlled animal facility with a 12-hour light cycle. All mouse experiments were performed in accordance with relevant guidelines and regulations. All animal experimental procedures were approved by the Committee of Animal Experimentation, Hiroshima University, and the Animal Care and Use Committee of the Tokyo Metropolitan Geriatric Hospital and Institute of Gerontology.

### Cell Lines and Culture Conditions

MC3T3-E1 cells (Sudo et al., 1983) were cultured in minimum essential medium Eagle alpha modification (α-MEM; M4526-500ML, Sigma-Aldrich, St. Louis, MO, United States) supplemented with 10% fetal bovine serum (FBS; Cambrex, East Rutherford, NJ, United States), 2 mM L-glutamate (G3126, Sigma-Aldrich), and 1% penicillin/streptomycin (P0781-100ML, Sigma-Aldrich) at 37°C in a humidified atmosphere of 5% CO_2_ in air. C3H10T1/2 cells (Reznikoff et al., 1973) were grown in Dulbecco’s modified Eagle’s medium (DMEM; D6429-500M, Sigma-Aldrich) supplemented with 10% FBS, 2 mM L-glutamate, and 1% penicillin/streptomycin at 37°C in a humidified atmosphere of 5% CO_2_ in air. MEFs were isolated from ICR and *ScxGFP* Tg mouse embryos at embryonic day (E)14.5. The head and visceral organs were removed from each embryo. The remainder of each body was minced using scissors and then digested with 0.1% trypsin (Difco)/0.1 mM EDTA (Dojin, Tokyo, Japan)/phosphate buffered saline (PBS) solution (3 ml per embryo) in a 50 ml tube at 37°C for 20 min. Dissociated MEFs were filtered through a 70 μm cell strainer (REF352360, BD Falcon, MA, United States), washed, and resuspended in DMEM supplemented with 10% FBS (Cell Culture Bioscience, Nichirei, Tokyo, Japan), 2 mM L-glutamate, and 1% penicillin/streptomycin. MEFs were seeded in culture dishes coated with 0.1% gelatin (G1890, Sigma-Aldrich) and grown at 37°C in a humidified atmosphere of 5% CO_2_ in air. For feeder cells, MEFs isolated from ICR mice were treated with 10 μg/ml mitomycin-C (M4287-2MG, Sigma-Aldrich). Tail tendons of *ScxGFP* Tg mice were isolated and placed in 60-mm cell culture dishes (3010-060, Iwaki, Fukushima, Japan) coated with native collagen from bovine dermis (IAC-30, Koken, Tokyo, Japan). Tenocytes outgrown from the tail tendons were grown in α-MEM supplemented with 10% FBS (Cell Culture Bioscience), 4 mM L-glutamine, and 50 µg/ml kanamycin at 37°C in a humidified atmosphere of 5% CO_2_ in air. The KY1.1 mouse ESC line, which is an F1 hybrid of C57BL/6J and 129S6/SvEvTac (Yagita et al., 2010) was kindly provided by Dr. Gen Kondoh, Kyoto University. *ScxGFP* iPSC lines were generated using Sendai virus vectors. Mouse ESCs and *ScxGFP* iPSCs were grown on feeder cells in 2i LIF medium at 37°C in a humidified atmosphere of 5% CO_2_ in air. The 2i LIF medium was comprised of 50% Neurobasal Medium (21103-049, Gibco, Carlsbad, CA, United States), 50% DMEM/F-12 + GlutaMAX-Ⅰ (10565-018, Gibco), 1% N2 Supplement (×100; 17502-048, Gibco), 2% B27 Supplement (×50; 17504-044, Gibco), 1 mM L-glutamine, 0.1 mM 2-mercaptoethanol (21985-023, Gibco), 1% penicillin/streptomycin, 3 mM CHIR99021 (CHIR; 1386, Axon Medchem, Reston, VA, United States), 1 mM PD0325901 (162-25291, Fujifilm Wako, Osaka, Japan), 1,000 unit/ml, and leukemia inhibitory factor (LIF; ESG1107. Sigma-Aldrich).

### Generation of *ScxGFP* iPSCs From *ScxGFP* Tg Embryos

*ScxGFP* iPSCs were generated using a Cytotune-iPS 2.0L Sendai Reprogramming kit (69020-81; ID Pharma, Tokyo, Japan) according to the manufacturer’s instructions. *ScxGFP* MEFs were seeded in gelatin-coated 6-well plates (3810-006, Iwaki) at a density of 5 × 10^5^ cells/well and cultured overnight in MEF medium. MEFs were then infected with Sendai virus vectors at a multiplicity of infection ranging from 1.25 to 5.0. Twenty-four hours after infection, the virus vectors were removed by replacing the medium with fresh MEF medium. Two days later, the medium was changed to 2i LIF medium. Six days after infection, the cells were subcloned onto feeder cells. Two weeks after infection, iPS colonies were picked and transferred onto feeder cells for expansion. Of the isolated 31 *ScxGFP* iPSC clones, three clones showing a clear round morphology were selected (*ScxGFP* iPSC-2, 18, and 20). Elimination of Sendai virus vectors and detection of *ScxGFP* transgene were checked by reverse transcriptase-polymerase chain reaction (RT-PCR) or genomic PCR using primer pairs specific for the virus vectors shown in Supplementary Table S1.

### Generation of Embryoid Body, Teratoma, and Chimeric Mice From *ScxGFP* iPSCs

For EB formation, *ScxGFP* iPSCs or mESCs were seeded in Costar 7007 ultra-low attachment 96 well round bottom culture plates (Corning, New York, NY, United States) at a density of 1 × 10^3^ cells/well. Cells were cultured in DMEM containing 15% FBS (Hana Nesco Bio, Tokyo, Japan), 0.1 mM 2-mercaptoethanol, 2 mM L-glutamate, and 1% penicillin/streptomycin (EB medium) for 7 days. For teratoma formation, trypsinized *ScxGFP* iPSCs and mESCs (0.5 or 1 × 10^6^ cells) were injected into the quadriceps femoris muscles and subcutaneously in the interscapular region of male SCID mice. Five weeks after injection, the mice were euthanized and teratomas were dissected. For toluidine blue staining, hematoxylin and eosin (H&E) staining, and immunofluorescent staining, the teratomas were fixed with 4% paraformaldehyde (PFA) in PBS, infiltrated with 20% sucrose in PBS, and then embedded in OCT compound (4583; Sakura Finetech Japan, Tokyo, Japan). To generate chimeric embryos, trypsinized *ScxGFP* iPSCs were microinjected into the perivitelline space of 8-cell embryos of ICR mice. These embryos were cultured in KSOM medium for 20 hours and transferred into the uterus of day 3 pseudo-pregnant ICR mice under anesthesia. *ScxGFP* Tg and chimeric embryos were collected at E13.5 and analyzed by fluorescence stereomicroscopy. Images were captured using a model MZ16FA stereomicroscope equipped with a model DFC7000T camera (Leica, Wetzlar, Germany).

### Tenogenic Differentiation of *ScxGFP* iPSCs

*ScxGFP* iPSCs and mESCs cultured on mitomycin-C-treated MEFs by using 2i LIF were trypsinized and seeded in gelatin-coated 24- or 48-well culture plates at a density of 4 or 2 × 10^4^ cells/well, respectively. Prior to mesodermal induction, iPSCs and mESCs were grown in DMEM-F-12 (DF) (10-090-CVR; Corning) supplemented with 5% FBS (Hana Nesco Bio), 2 mM L-glutamate, 1% penicillin/streptomycin, 10 ng/ml insulin (11 376 496 001; Roche, Basel Switzerland), 10 ng/ml transferrin (13366500; Roche), and 3 × 10^−8^ M sodium selenite (196-03192; Fujifilm Wako) (ITS) for 4 days. The medium was changed to the same DF-based medium containing 30 μM CHIR and 5 μM cyclopamine (Cyc; BML-GR344-0001; Enzo Life Sciences, Farmingdale, NY, United States) for mesodermal induction, and cultured for 4–5 days. After 1 or 2 days of culture in the same medium without CHIR and Cyc, the medium was changed to DF supplemented with 1% FBS, 2 mM L-glutamate, 1% penicillin/streptomycin, and ITS. The next day, the medium was changed to DF supplemented with 1% FBS, 2 mM L-glutamate, 1% penicillin/streptomycin, ITS, and 10 ng/ml recombinant human TGF-β2 (302-B2-010; R&D Systems Minneapolis, MN, United States). The cells were cultured for more than 9 days for tenogenic induction. Expression of paraxial and lateral plate mesoderm marker genes at T0 were analyzed using RT-PCR using the primer pairs shown in Supplementary Table S1. The PCR products were analyzed using the DNA-1000 kit for the microchip electrophoresis device MultiNA (292-27911-91; Shimadzu).

### Histological Staining

For histological analysis, cryosections of teratomas were stained with Gill’s hematoxylin (H-3401; Vector Laboratories, Burlingame, CA, United States) and 0.25% Eosin Y (058-00062; Fujifilm Wako). For toluidine blue staining, sections were stained with 0.05% toluidine blue O (T3260; Sigma-Aldrich) at pH 4.1. Control and induced tenogenic cells at T12 were fixed with 4% PFA in PBS for 30 min. The accumulation of collagen fibers, lipid drops, and cartilaginous matrix was stained with Picrosirius Red (PSR-1; Scy Tek Laboratories, Logan, UT, United States), Oil red O (154-02072; Fujifilm Wako), and Alcian blue 8GX (A3157; Sigma-Aldrich) adjusted at pH 1.0. Alkaline phosphatase (ALP) activity was detected using nitro-blue tetrazolium/5-bromo-4-chloro-3′-indolyphosphate (NBT/BCIP) (11681451001; Roche) staining. Calcification of cells was detected by Alizarin Red S (S5533; Sigma-Aldrich) staining.

### Immunofluorescent Staining

After post-fixation with 4% PFA in PBS for 5 min and washing with PBS, the teratoma sections were incubated with anti-Sox9 (AB5535; 1:800, Millipore, Billerica, MA, United States) and anti-GFP (GF090R; 1:1000, Nacalai Tesque, Kyoto, Japan) antibodies, diluted with 2% skim milk in PBS at 4°C overnight. Sections were then incubated with secondary antibodies conjugated with Alexa Fluor 488 (A-11006; 1:500, Invitrogen, Carlsbad, CA, United States) and 594 (A-11072; 1:500, Invitrogen). For immunofluorescent staining of cells, the cells were fixed with 4% PFA in PBS for 30 min in culture plates and washed with PBS. Incubation with primary and secondary antibodies was performed in the same way and under the same conditions as for the sections. The primary antibodies used for immunofluorescent staining of cells were anti-GFP, anti-pSmad3 (600-401-919; 1:250, Rockland Immunochemicals, Gilbertsville, PN, United States), anti-Sox9, anti-Tnmd (1:500) (Yoshimoto et al., 2017), anti-OCT4 (ab19857; 1:250, Abcam, Cambridge, United Kingdom), anti-SEAA1 (ab16285; 1:200, Abcam), anti-Nanog (ab80892; 1:200, Abcam), anti-SOX2 (ab97959; 1:500, Abcam), and anti-Aldh1a2 (HPA010022; Sigma-Aldrich). Secondary antibodies conjugated with Alexa Fluor 488 (A-11006 and A-11017; 1:500, Invitrogen) and 594 (A-11072; 1:500, Invitrogen) were used. Nuclei were counterstained with 4′,6-diamidino-2-phenylindole (DAPI; D9542; Sigma-Aldrich). Images were captured using a model DMRXA microscope equipped with a model DC500 camera (Leica), model IX70 microscope equipped with a model DP80 camera (Olympus, Tokyo, Japan), and BZ-X810 microscope (Keyence, Osaka, Japan).

### Quantitative Reverse Transcriptase-Polymerase Chain Reaction

Total RNA was extracted using the RNeasy Plus Mini Kit (74134; Qiagen, Hilden, Germany). Complementary DNA (cDNA) was synthesized from total RNA by using a PrimeScript RT Reagent Kit (RR037A: TaKaRa Bio, Shiga, Japan) according to the manufacturer’s instructions. RT-qPCR was performed using SYBR Premix Ex Taq II (RR820S; TaKaRa Bio) on a StepOne Real-Time PCR System (Thermo Fisher Scientific, Waltham, MA, United States) or ABI PRISM 7900HT (Applied Biosystems, Piscataway, NJ, United States). The thermal profiles for RT-qPCR were as follows: denaturation at 95°C for 30 s, followed by an amplification step with 40 cycles of 95°C for 5 s and 60°C for 34 s. The expression of target genes was normalized to that of ribosomal protein S18 (Rps18). Relative mRNA expression was calculated using the 2-ΔΔCT method. Each qPCR reaction was performed in triplicate. The primers used for RT-qPCR are shown in Supplementary Table S1.

### Quantitative Analysis for Chondrogenesis and Tenogenesis

To evaluate chondrogenesis and tenogenesis, cells at T11 were subjected to Alcian blue (AB) staining and immunofluorescence staining by using antibodies to Tnmd and GFP, respectively. To examine the percentage of Scx^+^ and Sox9^+^ cells in each well, cells at T3 were also subjected to immunofluorescence staining by using antibodies against Sox9 and GFP, respectively. Images of stained whole wells were captured using a model BZ-X810 microscope (Keyence); AB^+^, GFP^+^, and Tnmd^+^ areas were quantified using the Hybrid Cell Count Application (Keyence).

### FACS Analysis

For dissociation at T0, induced cells were treated with 0.05% trypsin-EDTA solution (T3924; Sigma-Aldrich) for 5 min at 37°C. Induced tenocytes at T4 and T13 were treated with 0.02% type II collagenase (CLSS-2; Worthington Biochemical, Lakewood, NJ, United States) for 5 min at 37°C and then treated with 0.05% trypsin-EDTA solution for 5 min at 37°C. Cells at T4 were directly analyzed using the LSRFortessa flow cytometer (BD Biosciences, Santa Clara, CA, United States). The dissociated cells at T0 and T13 were fixed and permeabilized using the Cyto-Fast Fix/Perm buffer (426803; BioLegend, San Diego, CA, United States) for 20 min at 25°C. After the cells were washed and resuspended in ×1 of the Cyto-Fast Perm Buffer, they were stained using anti-Tnmd primary antibody followed by R-phycoerythrin-conjugated donkey anti-rabbit IgG (711-116-152; Jackson Immunoresearch Laboratories, West Grove, PN, United States) for FACS analysis using a FACS Aria II instrument (BD Biosciences, Santa Clara, CA, United States).

### Cell Sorting for scRNA-Seq Analysis

The cells at T0 were digested with 0.05% Trypsin-EDTA at 37°C for 5 min. For T3, T6, or T9, we digested the cells with 0.05% Trypsin-EDTA at 37°C for 5–10 min after the digestion of the cells with 0.02% collagenase at 37°C for 3–5 min. We obtained single cells by pipetting the digested cell sheets. Dissociated cells were resuspended in washing buffer consisting of 2.5% FBS in PBS, counted, and stained with Hoechst 33342 (H-1399; Invitrogen) and SytoxRed dead-cell stain (S34859; Thermo Fisher Scientific). Hoechst positive/Sytox negative single cells were sorted into 1 µl of cell lysis buffer consisting of 0.3% NP40 (28324; Thermo Fisher Scientific), 0.12 dNTPs (N0477; New England BioLab, Ipswich, MA, United States), 1U RNase Inhibitor (2313A; TaKaRa Bio), and 0.11 µM 384 well-unique reverse transcription primer in a 348-well PCR plate (0030129547; Eppendorf, Hamburg, Germany).

### scRNA-Seq Library Preparation and Sequencing

scRNA-seq libraries were prepared by amplifying the 3′ untranslated region (UTR) of the transcripts by using the modified CEL-Seq2 protocol (Hashimshony et al., 2016), which replaced the SuperScript II reverse transcriptase with Maxima H minus (EP0752; Thermo Fisher Scientific), the second strand synthesis reagent with the second strand synthesis module (E6111; New England BioLabs). A total of 384 cells in the same plate were pooled after reverse transcription. Each condition was analyzed in triplicate. Sequencing reads were obtained from HiSeq 1500 platform at the following cycles: 15 cycles of Read1 (Unique Molecular Identifier: 6 bp, cell barcode: 9 bp) and 36 cycles of Read2 (Illumina, San Diego, CA, United States), as shown in Supplementary Table S2.

### scRNA-Seq Bioinformatic Analysis and Visualization

The raw BCL files were converted to FASTQ files by using Illumina bcl2fastq software. Adaptors (GATCGTCGGACT) and low-quality reads were trimmed using Trim Galore (v0.6.6). Reads were aligned to the mouse GRCm38 by using HISAT2(v2.2.1), and UMI count matrix was obtained using featureCounts (v2.0.1) and UMI-tools (v1.0.1). The genes that were detected in more than three cells and cells that express at least 200 genes were used for further analysis (4,592 of 4,608 cells). A total of 1146 cells at T0, 1151 cells at T3, 1144 cells at T6, and 1146 cells at T9 were analyzed. Two thousand genes were set as highly variable genes, and cell cycle-related effects were regressed by CellCycleScoring and SCTransform function in Seurat3.0 (Stuart et al., 2019). Dimensionality reduction, clustering analysis, visualization, and differentially expressed gene analysis among the clusters were also performed using the Seurat3.0 package with the following functions: RunPCA, FindNeighbors, FindClusters, RunUMAP, and FindMarkers with default parameters. In RunUMAP, the first 10 principal components (PCs) were used, and the fifth PC, which observed the batch effect, was excluded. phateR (v1.0.4) was used for visualization (Moon et al., 2019).

### Gene Set Enrichment Analysis and Gene Ontology

GSEA was performed for differentially expressed genes in cluster 8, compared to those in clusters 4, 5, and 7, by using clusterProfiler (v3.16.1) (Yu et al., 2012). R package with org.Mm.eg.db_3.11.4 was used.

### Analysis of the Effects of RA Signaling on Tenogenic Differentiation

After mesodermal induction, 1 μM of all-trans retinoic acid (R2625; Sigma-Aldrich) and BMS493 (3509; TOCRIS) were applied with or without TGF-β2 for 7 days. For investigating the effects of hedgehog signaling on the RA signaling-mediated suppression of GFP^+^ cell induction, 100 nM of smoothened agonist (SAG) (566660; Merck), an activator of hedgehog signaling, was used for treatment with or without TGF-β2 and ATRA.

### Statistical Analyses

The sample sizes were determined based on previously published work (Nakajima et al., 2018) and preliminary studies. The sample numbers and statistical analyses are described in the figure legends. Statistical significance was determined using GraphPad Prism 8 (GraphPad Software, San Diego, CA, United States). No statistical methods were used for the sample size determination. For comparisons of more than two means, one-way ANOVA followed by Dunnett’s test or Tukey’s post hoc test was employed. Significance values used in figures are *p < 0.05, **p < 0.01, ***p < 0.001, and ****p < 0.0001.

## Results

### Decreased Expression of Tenogenic Markers in Tenocytes Outgrown From Tail Tendons

**FIGURE 1:**
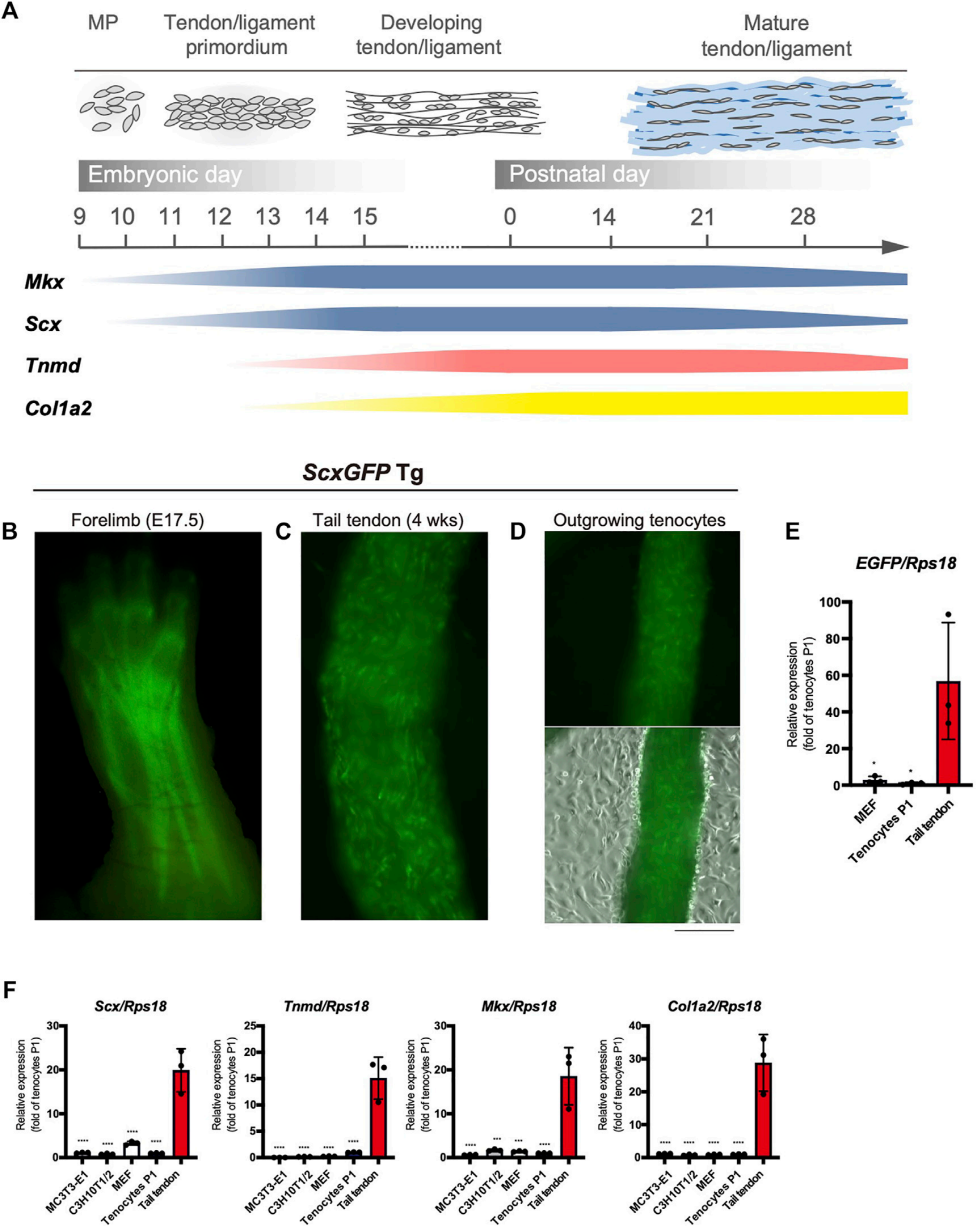
Decreased expression of tenogenic markers in tenocytes outgrown from tail tendons. **(A)** Schematic illustration of major tendon marker genes expressed in association with tendon development *in vivo*. Expression of *Mkx* and *Scx* are shown in blue. *Tnmd* and *Col1a2* expression are shown in pink and yellow, respectively. MP, mesenchymal progenitors. **(B)** A fluorescent image of the forelimb of *ScxGFP* embryo at E17.5. *Scx* expression visualized by GFP fluorescence is detected in the developing tendons and ligaments. **(C)** A fluorescent image of the dissected tail tendons from 4-week-old *ScxGFP* mice. Tenocytes of the tail tendon were positive for GFP expression. **(D)** Decreased GFP fluorescence in tenocytes outgrown from the *ScxGFP* tail tendons placed on type I collagen coated culture dish. On the surface of the tendon, outgrowing GFP^+^ tenocytes with a round morphology are observed. **(E)** Expression of *EGFP* in *ScxGFP* MEFs, cultured tenocytes of passage 1 (P1), and the tail tendon. Tenocytes and the tail tendon were isolated from 4-week-old *ScxGFP* Tg mice. *n* = 3. **(F)** Expression of tenogenic markers in MC3T3-E1 cells, C3H10T1/2 cells, MEFs, cultured tenocytes P1, and the tail tendon. *n* = 3. The data represent the mean ± SD. One-way ANOVA followed by Dunnett’s multiple comparison test. *p < 0.05, ***p < 0.001, ****p < 0.0001. Scale bar, 500 μm **(D)**.

The major tendon marker genes used in this study are shown in Figure 1A. During mouse embryogenesis, the expression of early tendon marker genes, *mohawk* (*Mkx*) and *Scx,* is initiated in the progenitors of the sclerotome and limb buds (Cserjesi et al., 1995; Anderson et al., 2006). These progenitors migrate and condense to form the tendon primordia in which tenocytes mature to express late marker genes, such as *Tnmd* (Shukunami et al., 2006; Shukunami et al., 2018), *Col1a1*, and *Col1a2* (Havis et al., 2014; Kelly et al., 2020) (Figure 1A). The tendons or ligaments of *ScxGFP* Tg mice were clearly visualized by green fluorescence (Figures 1B,C). Tenocytes attached to the surface of the tail tendon were positive for GFP, but GFP expression was dramatically decreased as outgrown tenocytes moved away from the tendon and migrated onto the culture dish (Figure 1D). We then examined the expression level of *enhanced green fluorescent protein* (*EGFP*) in cultured tenocytes at passage 1 (P1), mouse embryonic fibroblasts (MEFs), and tail tendons. Significantly decreased expression of *EGFP* was evident in the cultured tenocytes at P1 and in MEFs compared with that in the tail tendons (Figure 1E). The expression levels of tendon marker genes in these cultured cells were also significantly lower than those in the tail tendons (Figure 1F). Therefore, high levels of *in vivo* expression of tendon marker genes were not maintained even in primary and secondary tenocytes, suggesting that cell isolation from tissue triggers tenocyte dedifferentiation.

### Contribution of *ScxGFP* iPSCs to the Tendon/Ligament Lineage

**FIGURE 2:**
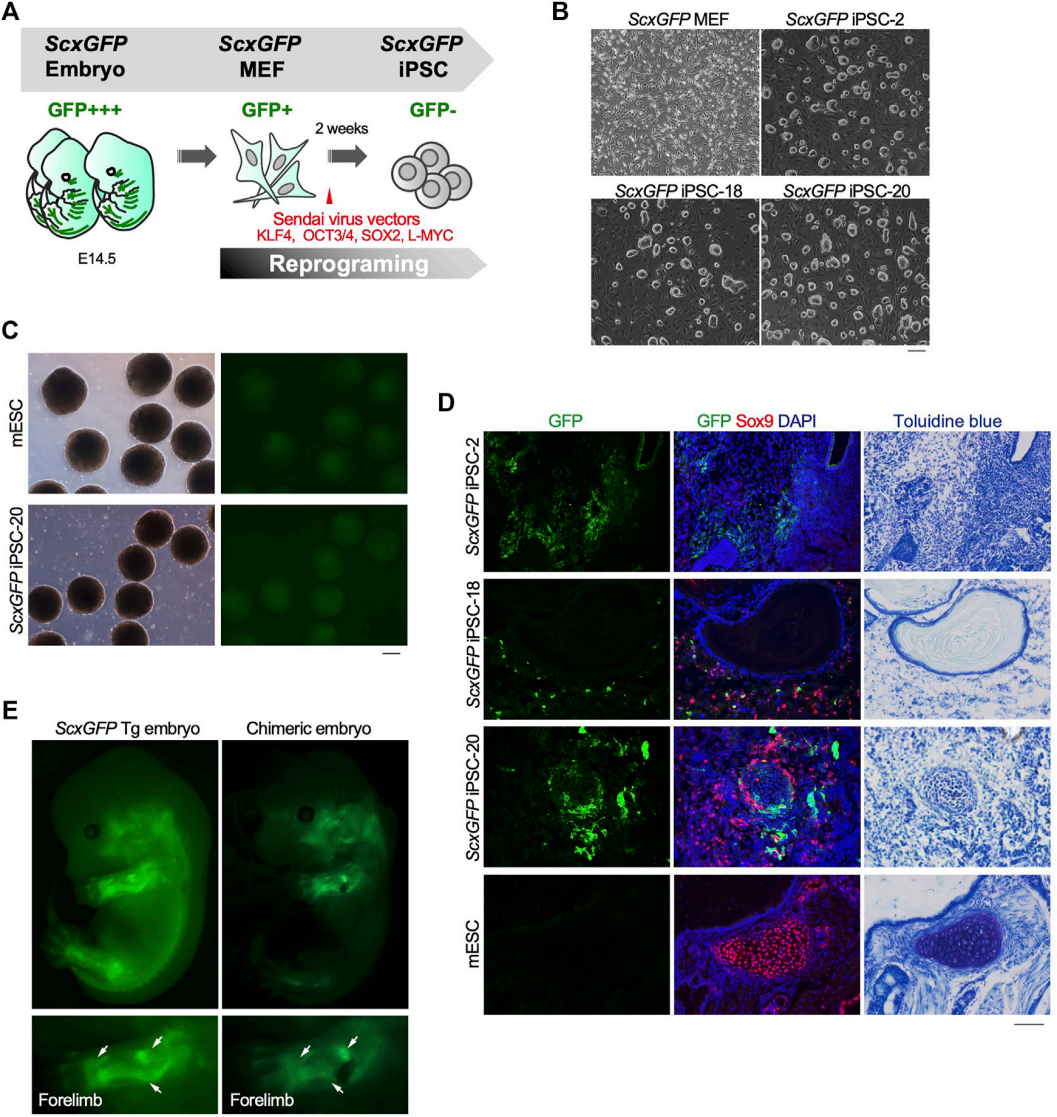
Contribution of *ScxGFP* iPSCs to the tendon/ligament lineage. **(A)** Schematic drawing of the generation of *ScxGFP* iPSCs by using Sendai virus vectors. **(B)** Phase contrast images of *ScxGFP* MEFs (P1), *ScxGFP* iPSC-2 (P20), -18 (P19), and -20 (P20) are shown. **(C)** Phase contrast and GFP fluorescent images of embryoid bodies generated by wild type mouse embryonic stem cells (mESC) and *ScxGFP* iPSC-20. **(D)** Immunostaining of GFP (green) and Sox9 (red) was performed on frozen sections prepared from teratomas formed from wild type mESCs and *ScxGFP* iPSCs-2, -18, and -20. On the far right, the images of toluidine blue staining of the corresponding region of the immunostained sections are shown. mESCs were used as negative control. **(E)** A *ScxGFP* Tg embryo at E13.5 and a chimeric mouse embryo generated using *ScxGFP* iPSC-20. Whole-body images of the left side and enlarged images of the left forelimb are shown. Scale bars, 200 μm **(B–C)** and 100 μm **(D)**.

The level of *EGFP* expression strongly correlated with that of each tendon marker gene in tenocytes isolated from *ScxGFP* Tg mice and tendon tissue (Figure 1). Taking advantage of GFP expression as an indicator of tenogenic differentiation, we sought to establish an *in vitro* tenogenic differentiation system by using *ScxGFP* Tg mice-derived cells. Considering the high reproducibility and stable maintenance of induced pluripotent stem cells (iPSCs), we used these cells as the starting material. iPSCs were generated by forced expression of *SOX2*, *OCT3/4*, *KLF4*, and *L-MYC* in *ScxGFP* MEFs by using Sendai virus vectors (Figure 2A). Three clones selected from the established iPSCs formed colonies with a round morphology similar to mouse ESC-like colonies (Figure 2B). Elimination of Sendai virus and the presence of the *ScxGFP* transgene were confirmed in the established *ScxGFP* iPSCs (Supplementary Figures S1A,B). Next, we evaluated the quality and pluripotency of the established clones of *ScxGFP* iPSCs. All *ScxGFP* iPSCs displayed high alkaline phosphatase (ALP) activity and expressed markers for ESCs (Supplementary Figures S2C,D). *ScxGFP* iPSCs could form embryoid bodies (EBs) and teratomas, including all three germ layers (Figure 2, Supplementary Figures S3E,F). GFP was not detected by fluorescent stereomicroscopy in *ScxGFP* EBs, but GFP^+^ cells were observed in teratomas of *ScxGFP* iPSCs (Figures 2C,D). GFP^+^ cells were mainly observed in the fibrous tissues of the teratomas (Figure 2D). We previously reported that *Scx* ^+^ cells appeared in Sox9^+^ areas, such as the sclerotome and limb buds, located around the cartilaginous region during musculoskeletal development (Sugimoto et al., 2013a). Likewise, GFP^+^ cells were found near Sox9^+^ cartilage in the teratoma (Figure 2D). Since teratomas of *ScxGFP* iPSC-20 contained a relatively large amount of bone or cartilaginous tissues closely related to the tendon and ligament lineage, we selected this clone for further analysis. To confirm *in vivo* tenogenic potential of iPSC clone, we generated chimeric embryos by using *ScxGFP* iPSC-20 (Figure 2E). GFP expression in a chimeric mouse was observed in the developing tendon and ligament primordia, with a pattern similar to that observed in a *ScxGFP* Tg mouse (Figure 2E). These results indicate that *ScxGFP* iPSCs faithfully express GFP in the tenogenic/ligamentogenic cell lineage and were, therefore, suitable for the establishment of an *in vitro* tenogenic differentiation system.

### Establishment of TGF-β2-Driven Tenogenic Differentiation System by Using *ScxGFP* iPSCs

**FIGURE 3:**
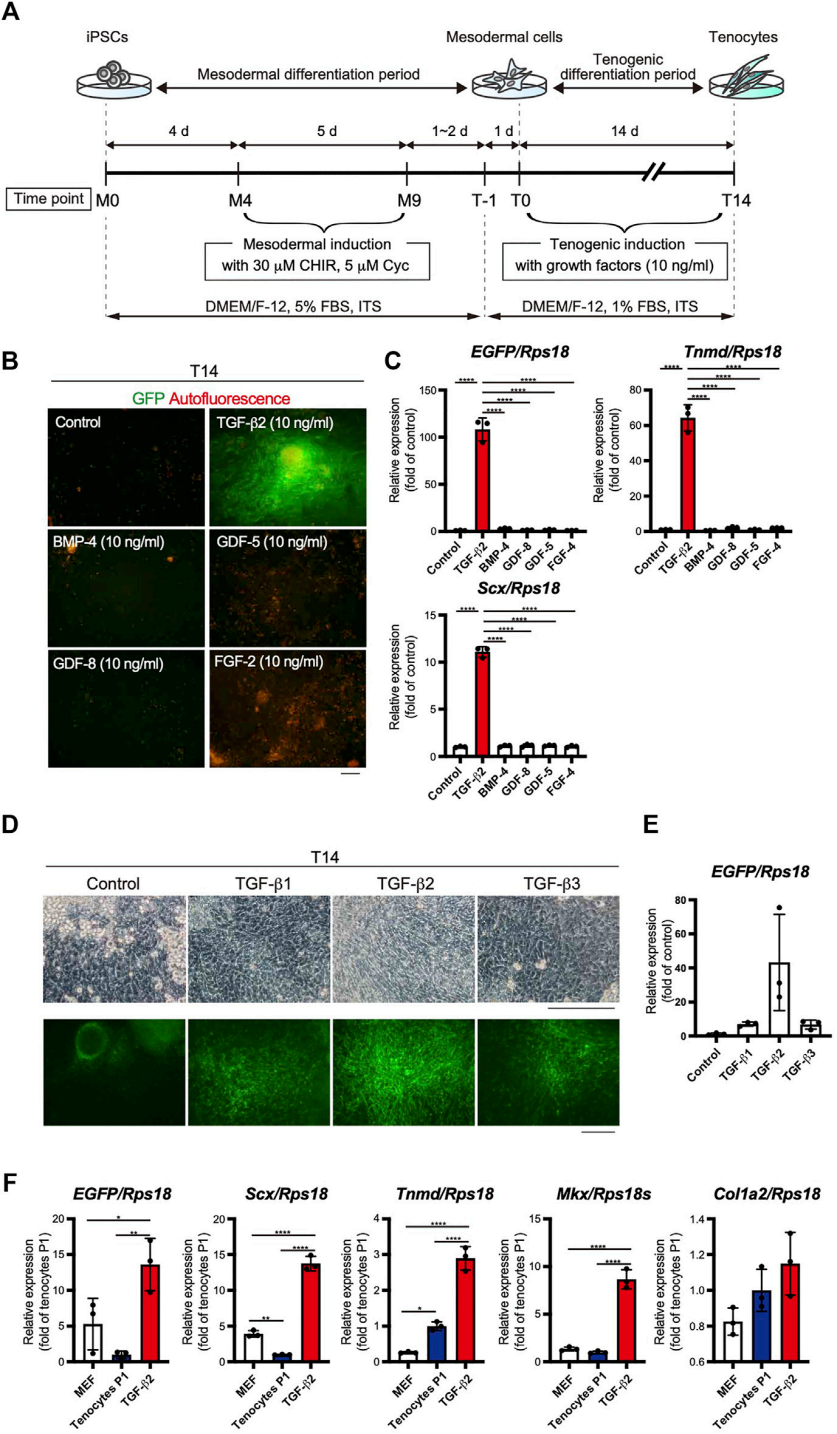
Optimization of the stepwise differentiation of *ScxGFP* iPSCs into tenocytes via mesodermal cells. **(A)** A schematic drawing showing the three-phase strategy for tenogenic differentiation from *ScxGFP* iPSCs. Time points during mesodermal induction and tenogenic induction are indicated as M0–M9 and T-1–T4, respectively. **(B)** Fluorescent images of cells treated with growth factors and control cells maintained without growth factors after mesodermal induction. Merged images of GFP signal (green) and autofluorescence (red) are shown. **(C)** The expression levels of *EGFP*, *Tnmd*, and *Scx* in cells shown in **(B)**. The relative expression of each gene is normalized to the control. *n* = 3. Mean Ct values are shown in Supplementary Table S3. **(D)** Phase contrast and green fluorescent images of cells maintained in the presence and absence (control) of TGF-β1, 2, and 3 for 14 days. **(E)** The expression level of *EGFP* in cells shown in **(D)**. *n* = 3. **(F)** The expression level of *EGFP*, and tenogenic markers in MEFs, cultured tenocytes P1, and cells treated with TGF-β2 at T14. *n* = 3. The data represent the mean ± SD. One-way ANOVA followed by Tukey’s multiple comparison test. *p < 0.05, **p < 0.01, ****p < 0.0001. Scale bars, 100 μm **(B)** and 200 μm **(D)**.

The use of EBs formed from iPSCs for tenogenic differentiation has been previously reported (Komura et al., 2020). However, EB-based methods are cumbersome, and it is difficult to uniformly control the properties of cells contained in EBs. Therefore, we focused on a stepwise differentiation method in monolayer culture. The Wnt agonist CHIR99021 (CHIR), in combination with the Hedgehog inhibitor cyclopamine, has been used to induce mesodermal cells from pluripotent ESCs (Kanke et al., 2014). According to this study, we first induced mesodermal cells from *ScxGFP* iPSCs maintained in the presence of CHIR and cyclopamine. We then examined the effects of several growth/differentiation factors on GFP expression to induce Scx^+^ cells. The whole process consisted of mesodermal induction (M0-M9) for 9 days, followed by recovery for 1 or 2 days, pre-conditioning for 1 day, and tenogenic induction (T0-T14) for 14 days (Figure 3A). The growth factors that were selected have been reported to be involved in tendon development. The factors used included TGF-β2 or 3 (Pryce et al., 2009), TGF-β1 (Yin et al., 2016a), bone morphogenetic protein-4 (BMP-4) (Blitz et al., 2009), GDF-5 (Tan et al., 2012), GDF-8 (Mendias et al., 2008), and FGF-2 (Tokunaga et al., 2015; Hyun et al., 2017). TGF-β2 induced the most potent expression of GFP and tendon marker genes (Figures 3B,C). All isoforms of TGF-β (TGF-β1-3) were able to induce Scx^+^ cells, but TGF-β2 was most effective in GFP expression during differentiation of *ScxGFP* iPSC-derived mesodermal cells (Figures 3D,E). When the expression levels of tenogenic markers in cells treated with TGF-β2 were compared with those of MEFs and cells outgrown from the tendon (Figure 3F), significantly higher expression of these markers was detected in TGF-β2 treated cells, except for *Col1a2*. We speculate that since our system is two-dimensional, it may not achieve the high level of type 1 collagen expression required for *in vivo* tissue construction. We also tested the effect of TGF-β2 on *in vitro* tenogenic differentiation based on EB formation. GFP expression was very weak (Supplementary Figure S2). The results indicated the successful establishment of efficient tenogenic differentiation induced by TGF-β2 after mesodermal induction from *ScxGFP* iPSCs by using CHIR and cyclopamine.

### Inverse Relationship Between TGF-β2-Driven Tenogenic Differentiation and Chondrogenic Differentiation

**FIGURE 4:**
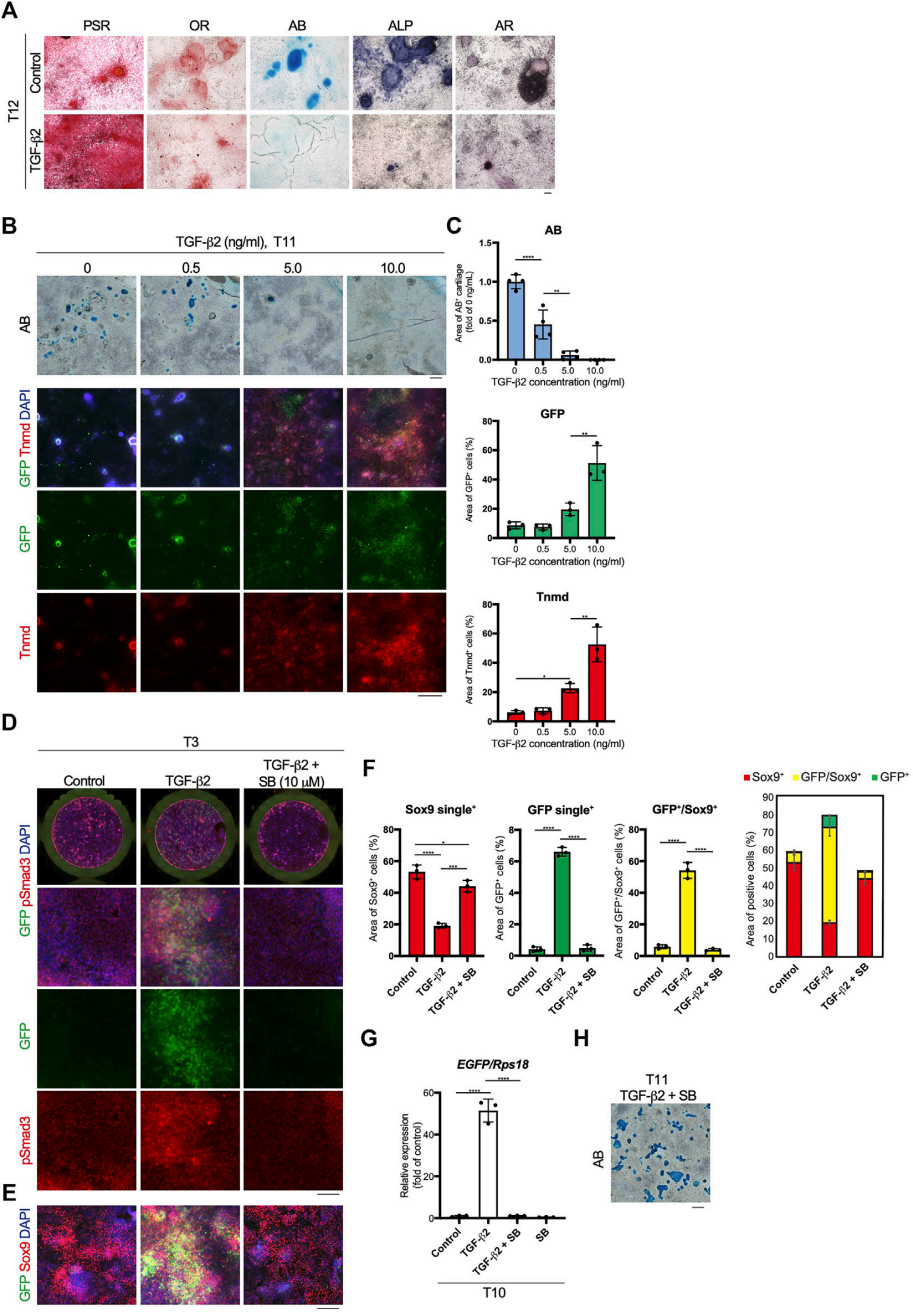
Inverse relationship between TGF-β2-dependent tenogenic and chondrogenic differentiation. **(A)** Staining with Picrosirius Red (PSR), Oil red O (OR), Alcian blue (AB), alkaline phosphatase (ALP), and Alizarin red (AR) in control and cells treated with TGF-β2 at T12. **(B)** Dose-dependent effects of TGF-β2 on tenogenic and chondrogenic differentiation. AB staining or immunofluorescent staining for GFP and Tnmd in cells treated with various concentrations of TGF-β2 at T11 are shown. **(C)** Quantitative analysis of AB (*n* = 4), GFP^+^ (*n* = 3), and Tnmd^+^ (*n* = 3) areas. **(D)** Immunofluorescent staining for GFP and phosphorylated Smad3 (pSmad3) of control, TGF-β2-treated, and TGF-β2 and SB431542 (SB)-treated cells at T3. Merged images of the whole culture wells (top panels) and magnified images (lower panels) are shown. **(E)** Immunofluorescent staining for GFP and Sox9 of control, TGF-β2-treated, and TGF-β2 and SB-treated cells at T3. **(F)** Quantitative analysis of GFP^+^ (*n* = 3), GFP^+^/Sox9^+^ (n = 3), and Sox9^+^ (*n* = 3) areas. The combined graph of each area is shown on the far right. **(G)** The expression levels of *EGFP* in control and cells treated with TGF-β2, TGF-β2 with SB, and SB at T10. *n* = 3. **(H)** Chondrogenic differentiation of cells treated with TGF-β2 and SB at T11. Cartilaginous areas were stained with AB. The data represent the mean ± SD. One-way ANOVA followed by Tukey’s multiple comparison test. *p < 0.05, **p < 0.01, ****p < 0.0001. Scale bars, 100 μm **(A and D)**, 200 μm **(E)**, and 500 μm **(B and H)**.

Mesodermal progenitors are multipotent and differentiate into mesenchymal cells, including tenocytes and ligamentocytes. We detected the accumulation of type I or III collagen fibers stained with Picrosirius Red (PSR), lipids stained with Oil red O (OR), cartilage nodules stained with Alcian blue (AB), osteogenic regions showing ALP activity, and calcified regions stained with Alizarin red (AR) in control wells at T12 (Figure 4A). Upon tenogenic induction by treatment with TGF-β2, type I or III collagen fibers copiously accumulated in the culture, whereas the formation of AB-stained cartilaginous nodules was completely suppressed (Figure 4A). Notably, chondrogenic differentiation was observed in all wells, except for those treated with TGF-β2 (Supplementary Figure S3). We further examined the dose-dependent effects of TGF-β2 on chondrogenic and tenogenic differentiation. With increasing concentrations of TGF-β2, the AB-stained cartilaginous areas decreased, while GFP or Tnmd^+^ tenogenic areas increased (Figures 4B,C). During limb tendon development, TGF-β signaling is mediated by the intracellular phosphorylation of Smad2/3 (Havis et al., 2014). Phosphorylation of Smad3 was observed in GFP^+^ cells treated with TGF-β2 at T3. In contrast, pSmad3 was not detected in GFP-negative cells in the control well or in cells treated with both TGF-β signal inhibitor SB431542 (SB) and TGF-β2 (Figure 4D). While the addition of TGF-β2 increased the number of GFP^+^ cells and GFP^+^/Sox9^+^ cells, many Sox9^+^ cells were observed among the control cells or the cells treated with TGF-β2 and SB (Figure 4F). *EGFP* expression at T10 was almost completely suppressed by the inhibition of TGF-β signaling (Figure 4G). Conversely, the inhibition of endogenous and exogenous TGF-β led to chondrogenic differentiation (Figures 4G,H). These results clearly indicated an inverse relationship between tenogenesis and chondrogenesis regulated by TGF-β signaling.

### Dynamics of Tenogenic Differentiation Induced by TGF-β2

**FIGURE 5:**
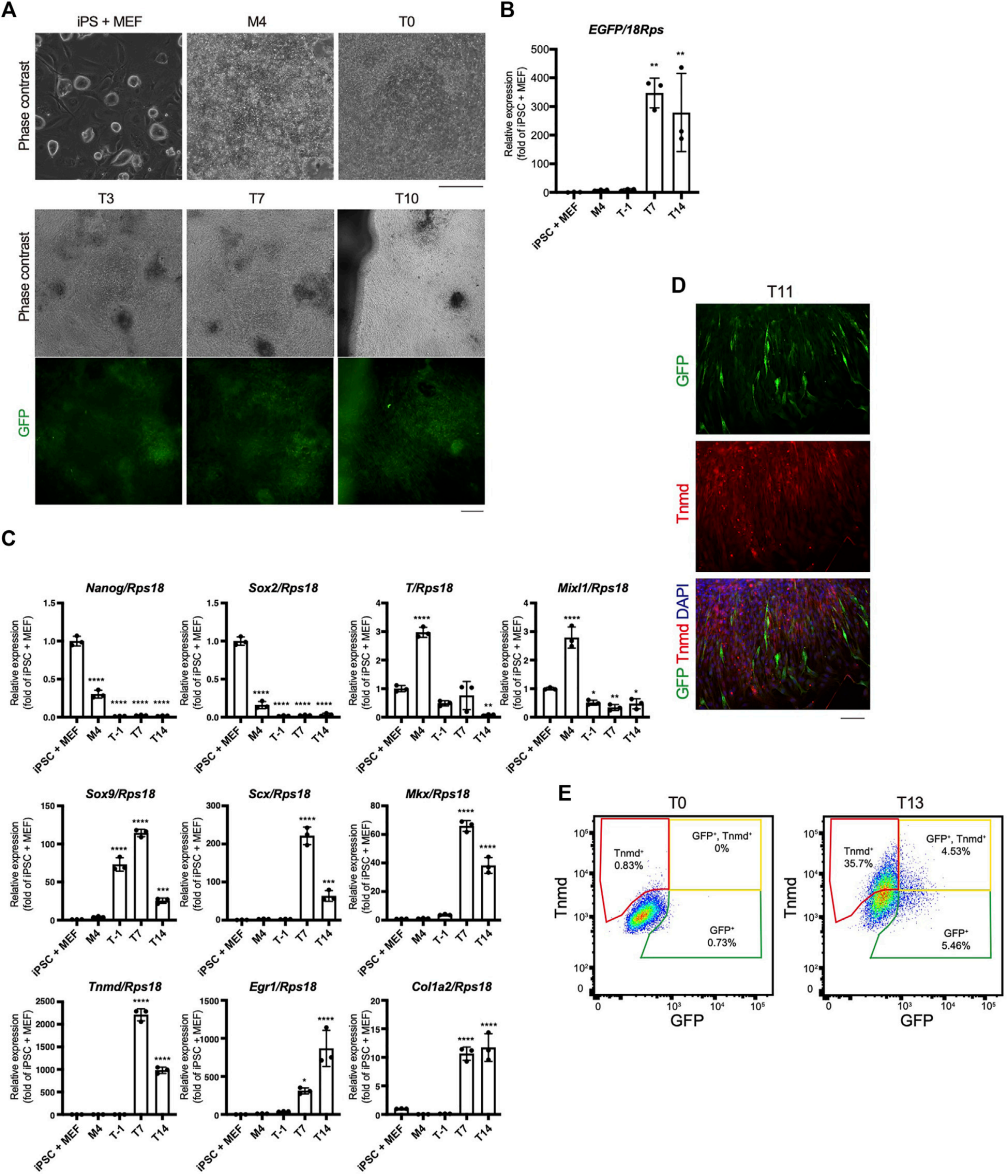
Time course of *in vitro* tenogenic differentiation induced by treatment with TGF-β2. **(A)** Cell morphologies and GFP expression during *in vitro* differentiation from *ScxGFP* iPSCs. **(B)** *EGFP* expression in iPSCs and induced cells at M4, T-1, T7, and T14. *n* = 3. **(C)** The expression levels of marker genes for ESCs (*Nanog*, *Sox2*), mesoderm (*T* and *Mixl1*), sclerotome (*Sox9*), and tendon (*Scx*, *Mkx*, *Tnmd*, *Egr1*, and *Col1a2*) during tenogenic differentiation. *n* = 3. Mean Ct values are shown in Supplementary Table S3. **(D)** Immunofluorescent staining for GFP (green) and Tnmd (red) of induced cells at T11. **(E)** FACS analysis of induced cells at T0 and T13. Green, yellow, and red polygonal regions indicate GFP^+^, GFP^+^/Tnmd^+^, and Tnmd^+^ tenogenic cells, respectively. The data represent the mean ± SD. One-way ANOVA followed by Dunnett’s multiple comparison test. iPSC + MEFs was used as a control group. *p < 0.05, **p < 0.01, ***p < 0.001, ****p < 0.0001. Scale bars, 200 μm (**A)** and 100 μm **(D)**.

The time course of tenogenic differentiation was examined next. The cell morphology changed markedly during induction by TGF-β2. Green fluorescence was clearly detected at T3 and was maximum at T7 (Figure 5A). *EGFP* expression was correlated with the intensity of green fluorescence (Figures 5A,B). The expression levels of genes related to pluripotency—*Nanog homeobox* (*Nanog*) and *SRY-box 2* (*Sox2*)—were highest in iPSCs and decreased with mesodermal induction (Figure 5C). Expression of the mesodermal marker genes *brachyury* (*T*) and *Mixl1* was highest at M4 (Figure 5C). *Sox9* expression was elevated at T1 just prior to tenogenic induction (Figure 5C), consistent with the expression during axial and appendicular tendon development (Sugimoto et al., 2013a). The *Scx*, *Mkx*, *Tnmd*, *early growth response 1* (*Egr1*), and *Col1a2* tenogenic markers were dramatically upregulated upon tenogenic induction (Figure 5C). *Egr1* is a zinc finger transcription factor that positively regulates the expression of *Col1a1* (Lejard et al., 2011). Interestingly, the expression of *Scx*, *Mkx*, and *Tnmd* decreased after T7, while *Egr1* was upregulated until T14 (Figure 5C). The decreased expression of tenogenic marker genes during postnatal tendon maturation has been demonstrated (Yin et al., 2016b; Grinstein et al., 2019). Thus, these gene expression dynamics suggest further maturation of tenocytes at a later time point, although we cannot exclude the possibility that non-tenogenic cells were increased from T7 to T14. Immunofluorescent staining for GFP and Tnmd at T11 revealed the presence of a mixed population of Scx^+^, Scx^+^/Tnmd^+^, and Tnmd^+^ cells (Figure 5D). At T13, fluorescence-activated cell sorting (FACS) analysis revealed a significant increase in the number of Scx^+^, Scx^+^/Tnmd^+^, and Tnmd^+^ cells compared to T0 when the cells were not subjected to tenogenic induction (Figure 5E). The obvious expression of Tnmd protein provided additional evidence of tenocyte maturation, although the induced tenocytes were a heterogeneous population, at least in terms of Scx and Tnmd expression.

### Identification of Mature Tenocyte Population Induced by TGF-β2 in *ScxGFP* iPSC Culture by scRNA-Seq

**FIGURE 6:**
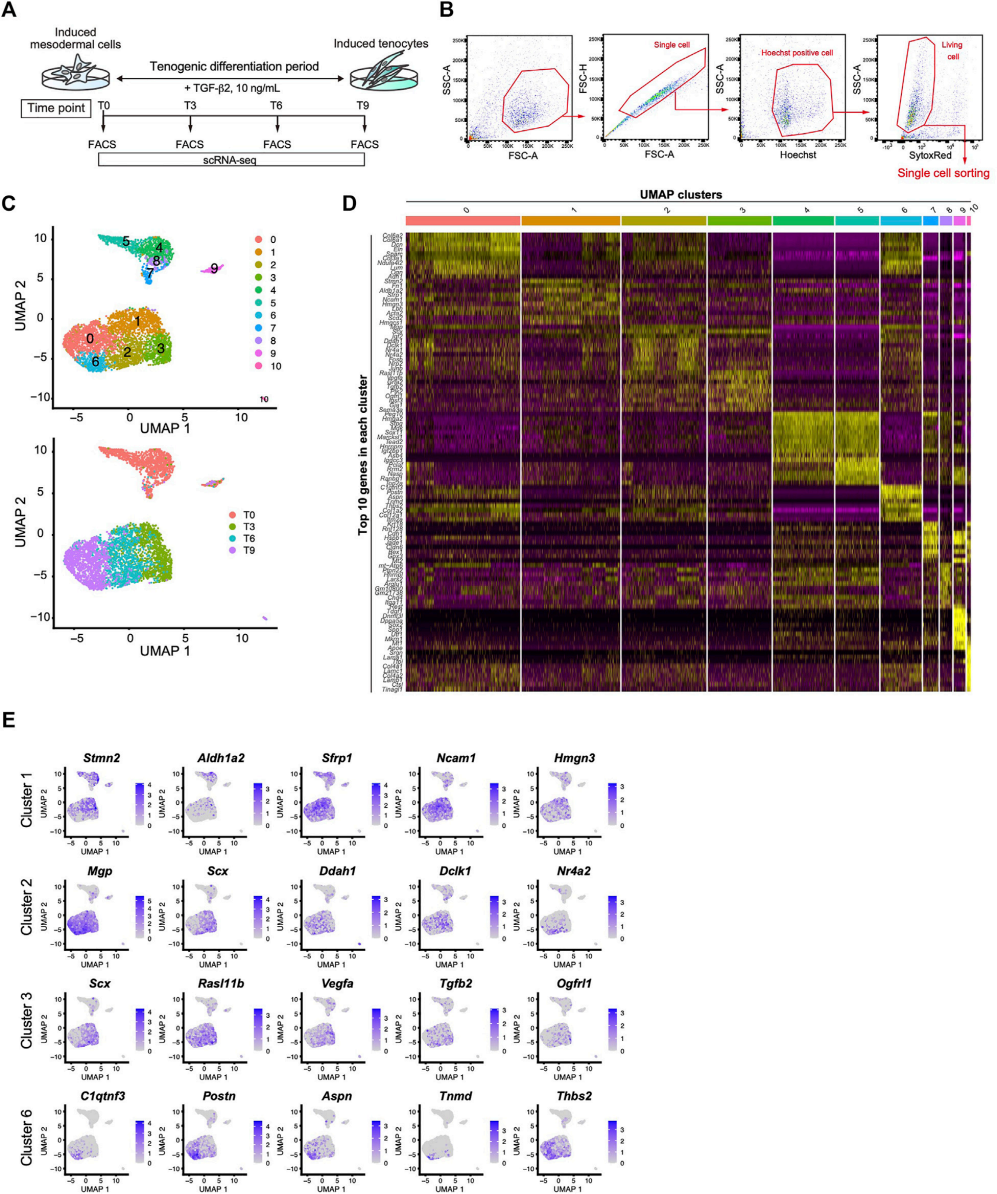
Identification of the mature tenocyte population in the TGF-β2-induced *ScxGFP* iPSC culture by Single-cell RNA sequencing. **(A)** Time schedule for cell isolation by FACS. Cells isolated by trypsinization were subject to FACS at T0 (before TGF-β2 treatment), T3, T6, and T9. **(B)** Gating strategy for single-cell sorting. Hoechst^+^, doublet- and dead cell-excluded cells were sorted to 384 well plates. **(C)** UMAP plot generated from data of 4,592 cells. Upper panel shows 11 distinct clusters. Lower panel shows each time point displayed on UMAP plot. **(D)** A heatmap showing the expression of the top 10 representative genes in each UMAP cluster. **(E)** UMAP plots showing the expression of the top five representative genes in cluster 1, 2, 3, and 6. Expression levels are expressed by blue to gray gradient color.

We next performed scRNA-seq to further clarify the tenogenic differentiation process. Single cell sorting was performed during tenogenic differentiation and a total of 4,592 cells were analyzed (Figures 6A,B). Uniform manifold approximation and projection (UMAP) clustering revealed 11 distinct clusters (Figure 6C, upper panel). A gradual temporal change was clearly evident when the differentiation time points were displayed on the UMAP plot (Figure 6C, lower panel). Cluster 9 included the pluripotent marker genes *Tdgf1* (Minchiotti, 2005), *Utf1* (Okuda et al., 1998), and *Sox2* (Avilion et al., 2003), indicating that the cells remained undifferentiated (Figure 6D and Supplementary Figure S4). Cluster 10 appeared to be endothelial cells because this cluster was characterized by the expression of endothelium-associated genes that included *Srgn* (Kulseth et al., 1999), *Tfpi* (Ameri et al., 1992), and basement membrane genes (Figure 6D and Supplementary Figure S4). We did not examine these clusters any further, and focused on the tenogenic differentiation pathway. Cluster 1 included *Aldh1a2* and *Acta2* (Figures 6D,E, and Supplementary Figure S4). Aldh1a2 is a major retinoic acid (RA) generating enzyme that has been reported in tendon precursors of chicken embryos (Berggren et al., 2001). Although *Acta2* is not expressed in tenocytes, lineage tracing studies have demonstrated that tenocytes are derived from *Acta2* ^+^ progenitors during postnatal growth (Dyment et al., 2014). Therefore, cluster 1 appears to represent early tenogenic progenitors. *Scx* ^+^ cells were enriched in clusters 2 and 3, and *Tgfb2* was enriched in cluster 3. The findings indicated that clusters 2 and 3 represent differentiating tenogenic cells (Figures 6D,E, and Supplementary Figure S4). Given that tenocytes are derived from Scx^+^/Sox9^+^- or Scx^+^/Sox9^-^-lineage cells, whereas ligamentocytes are descendants of Scx^+^/Sox9^+^-lineage cells (Sugimoto et al., 2013a; Huang et al., 2019), we examined the expression status of *Scx* and *Sox9*. Upon treatment with TGF-β2, the *Scx* ^*+*^ */Sox9* ^*+*^ cell population expanded significantly in association with the marked increase in the *Scx* ^*+*^ cell population, while the *Sox9* ^*+*^ cell population was diminished (Figure 4F and Supplementary Figure S5). These results provided evidence that our induction protocol can generate tenogenic and ligamentogenic progenitor populations, allowing monitoring of the tendon/ligament formation process during development. Among the UMAP clusters, cluster 6 was notable because it was highly enriched for known tendon genes. The top five enriched genes in cluster 6 were highly upregulated during tendon development (Figure 6E) (Havis et al., 2014). Thus, cluster 6 is likely a mature tenocyte population. Although cluster 0 is in close proximity to cluster 6, the expression levels of *Scx* in cluster 0 were low compared with those in cluster 2 (Figure 6E). Therefore, tenogenic differentiation appears to proceed primarily through cluster 2 and cluster 0 seems to be enriched in general fibroblasts.

### scRNA-Seq Reveals Tenogenic Differentiation Trajectory

**FIGURE 7:**
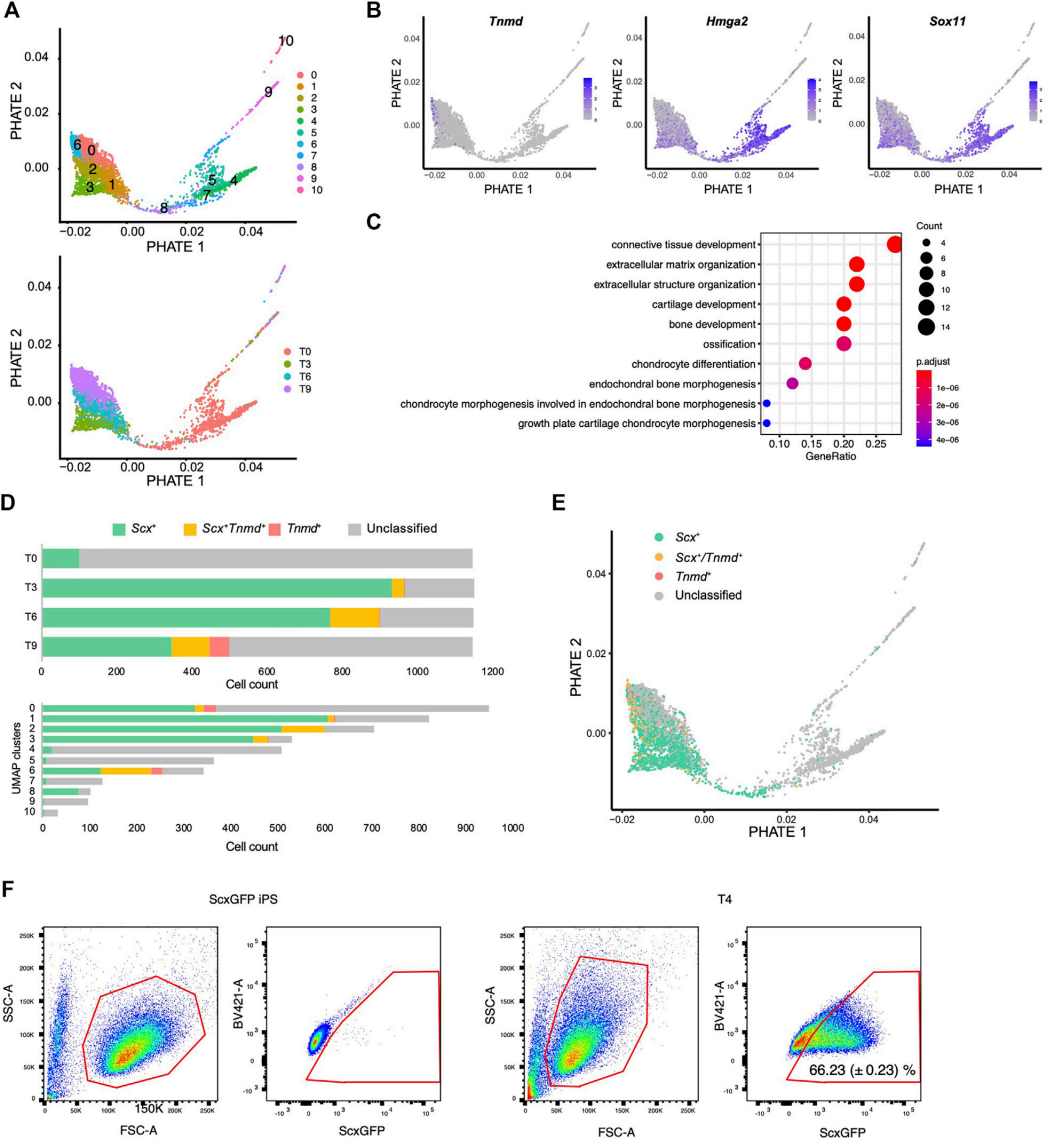
Single-cell RNA sequence analysis reveals the tenogenic differentiation trajectory. **(A)** PHATE plots displaying 11 UMAP clusters (upper) and each time point (lower). **(B)** PHATE plots showing *Tnmd*, *Hmga2*, and *Sox11* expression. The expression levels are expressed by blue to gray gradient color. **(C)** GO analysis for top 50 genes enriched in a cluster 8 against cluster 4, 5, and 7. **(D)** Number of *Scx* ^+^ (green), *Scx* ^+^/*Tnmd* ^+^ (double^+^, yellow), and *Tnmd* ^+^ (red) cells in each time point (upper) or UMAP clusters (lower) is shown. **(E)** PHATE plot showing *Scx*/*Tnmd* expression status. **(F)** Induction efficiency of GFP^+^ cells, as investigated by FACS analysis. *ScxGFP* iPS cells were used as the negative controls (left two panels). T4 cells undergoing differentiation induction were used for the analysis (right two panels). The data represent the mean SD (*n* = 3). The red polygons enclose the analyzed cells.

We next used another dimensionality reduction method, the potential of heat diffusion for affinity-based transition embedding (PHATE) (Moon et al., 2019), to accurately visualize the data structure of the differentiation pathway. When UMAP clusters were displayed on the PHATE plot, two end points were clearly revealed with cluster 6 located at one edge and cluster 4 located at another edge (Figure 7A). The edge of cluster 6 was highlighted by *Tnmd*, whereas the edge with cluster 4 was highlighted by *Hmga2* and *Sox11* expression (Figure 7B and Supplementary Figure S4). These genes have been reported as characteristic transcription factors of MSCs (Kubo et al., 2009). The findings provided evidence that the edges with cluster 4 and cluster 6 represent the beginning and end, respectively, of the differentiation pathway. We also noticed that cluster 8 bridged the gap between the T0 cluster and the T3-T9 cluster that was disconnected in the UMAP plot (Figure 7A lower panel and 6C). Gene ontology (GO) analysis indicated that cluster 8 was enriched for genes categorized in the GO terms of connective tissue structure and extracellular matrix organization (Figure 7C). The expression status of *Scx* and *Tnmd* was examined to better understand the progression of tenogenic differentiation. Induced cells first became *Scx* ^+^ followed by the emergence of *Scx* ^+^/*Tnmd* ^+^ cells and *Tnmd* ^+^ cells appeared lastly (Figure 7D, upper panel). This progression is consistent with *in vivo* tendon development, where Scx acts as an upstream transcriptional activator of Tnmd (Shukunami et al., 2006; Shukunami et al., 2018) and its expression diminishes as the tendon matures (Yin et al., 2016b; Grinstein et al., 2019). Interestingly, most of cluster 8 consisted of *Scx* ^+^ cells, unlike the other T0 components, that is, clusters 4, 5, and 7 (Figure 7D, lower panel). These findings suggest that cluster 8 represents the transition population toward the tenogenic lineage. When *Scx*/*Tnmd* expression status was displayed on the PHATE plot, a clear differentiation trajectory was observed (Figure 7E). The results of scRNA-seq also showed that the number of *Scx* ^*+*^ cells was maximal at T3 (Figure 7D). Therefore, we waited for 24 h for the accumulation of the GFP protein and examined the maximum induction efficiency of GFP^+^ cells using FACS at T4. In T4, 66.23% of the cells were GFP^+^ (Figure 7F).

### Retinoic Acid Negatively Regulated Induction of Scx^+^ Cells Possibly Through a Cell Autonomous Manner

**FIGURE 8:**
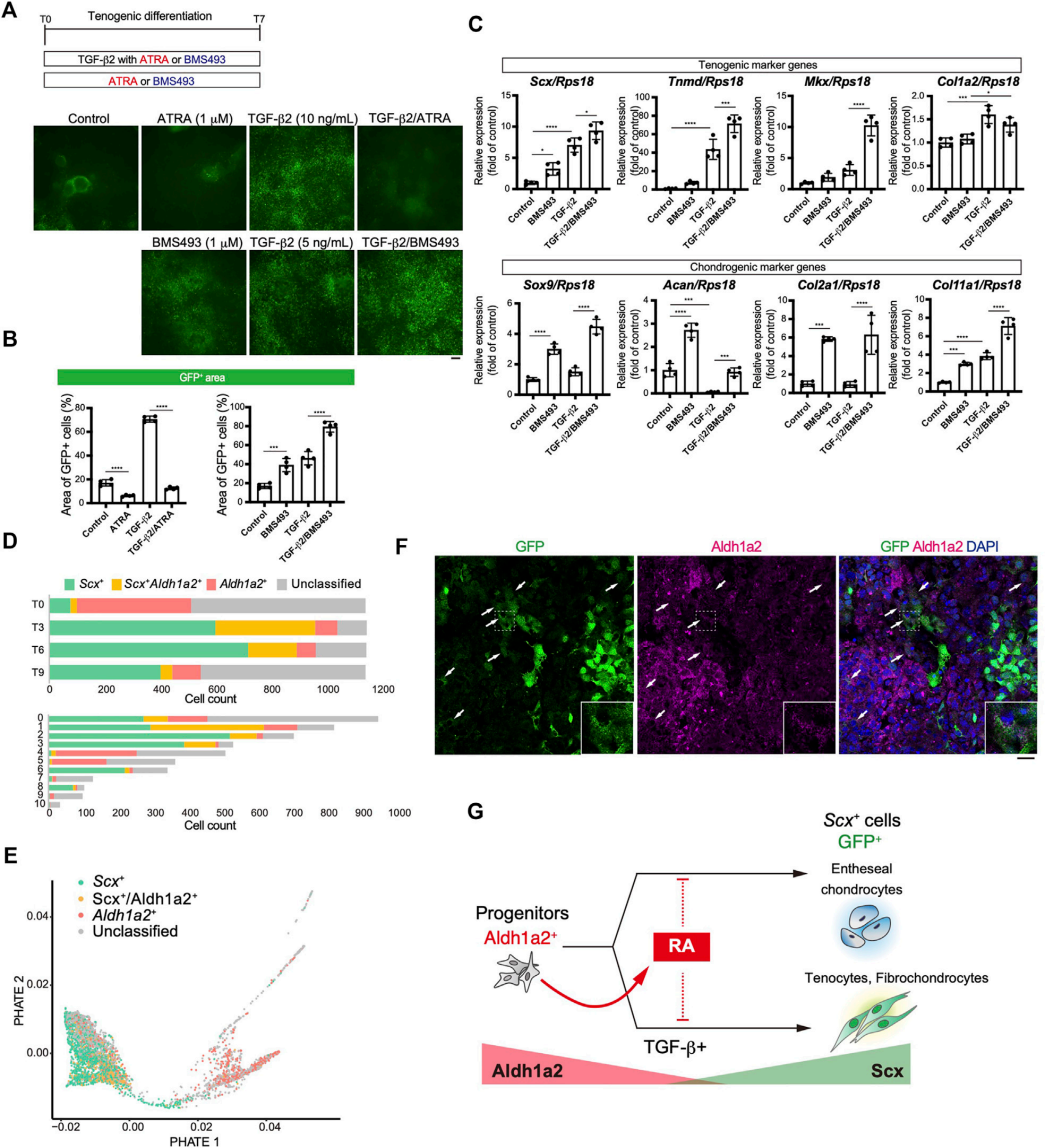
Retinoic acid negatively regulated induction of Scx^+^ cells. **(A, B)** Effects of retinoic acid signaling on tenogenic induction were investigated by using all-trans retinoic acid (ATRA) and BMS493 with or without TGF-β2. Immunofluorescent staining for GFP at T7 **(A)** and percentage of GFP^+^ areas **(B)** in each condition were presented. **(C)** Expression level of tenogenic and chondrogenic marker genes at T7 were shown. The data represent the mean ± SD (*n* = 4). One-way ANOVA followed by Dunnett’s multiple comparison test. **(D)** Number of *Scx* ^*+*^ (green), *Scx* ^*+*^ */Aldh1a2* ^*+*^ (double^+^, yellow), and *Aldh1a2* ^*+*^ (red) cells in each time point (upper) or UMAP clusters (lower) is shown. **(E)** PHATE plot showing *Scx*/*Aldh1a2* expression status. **(F)** Immunofluorescent staining for GFP and Aldh1a2 at T4 cells. Arrows indicate GFP^+^/Aldh1a2^+^ cells. Scale bar: 25 μm. **(G)** Illustration of a RA effect on GFP^+^ cell induction and the relation between GFP^+^ and Aldh1a2^+^ cells. Aldh1a2 and GFP expression level were shown as red and green triangle. *p < 0.05, ***p < 0.001, ****p < 0.0001.

We focused on retinoic acid (RA) signaling because of specific *Aldh1a2* expression in early tenogenic progenitors (cluster1) (Figure 6E). To examine the effects of RA signaling during tenogenic differentiation, we used all-trans retinoic acid (ATRA) and BMS493, a pan-retinoic acid receptor inverse agonist, in the presence or absence of TGF-β2. Upon addition of ATRA, GFP^+^ areas were significantly reduced compared with those of control (Figures 8A,B). ATRA also suppressed the induction of GFP^+^ cells by TGF-β2 to basal levels (Figures 8A,B). In contrast, even in the absence of TGF-β2, BMS493 increased GFP^+^ areas (Figures 8A,B). BMS493 alone upregulated *Scx* and all chondrogenic markers including *Sox9* but other tenogenic markers were not induced (Figure 8C), suggesting the induction of entheseal chondrocytes derived from Scx^+^/Sox9^+^ cells (Sugimoto et al., 2013a). More GFP^+^ cells were induced by adding BMS493 in the presence of TGF-β2 (Figure 8B). TGF-β2 alone upregulated early tenogenic markers (*Scx*) as well as a late tenogenic marker (*Tnmd and Col1a2*). Interestingly, both tenogenic (*Scx*, *Tnmd*, and *Mkx*) and chondrogenic (*Sox9*, *Acan*, *Col2a1*, and *Col11a1*) markers were significantly upregulated when BMS493 and TGF-β2 were added (Figure 8C). Since ATRA has been reported to suppress fibrocartilage differentiation (Kaji et al., 2021) which is characterized by induction of cells simultaneously expressing *Col1* and *Col2* but negative for *Tnmd* (Benjamin and Evans, 1990; Sugimoto et al., 2013a), our results suggest that both tenogenic and fibrocartilaginous chondrogenic differentiation are negatively regulated by activation of RA signaling. We next investigated the relation between endogenous RA signaling and tenogenic/chondrogenic differentiation by analyzing the expression status of *Scx* and *Aldh1a2* in our single cell analysis. At T0, most of *Aldh1a2* ^+^ cells were negative for *Scx*, but expansion of *Scx* ^+^/*Aldh1a2* ^+^ cells was observed at T3 and then *Scx* ^+^ cells became dominant in association with decrease of *Scx* ^+^/*Aldh1a2* ^+^ cells (Figure 8D, upper panel). The larger *Aldh1a2* ^+^ populations were observed in MSC like cluster 4 and 5, whereas mature tenocyte population (cluster 6) was predominantly composed of *Scx* ^+^ cells (Figure 8D, lower panel). Displaying the *Scx*/*Aldh1a2* expression status on the PHATE plot, a clear differentiation trajectory was observed (Figure 8E). Double immunostaining of cells at T4 revealed that GFP^+^, Aldh1a2^+^, and GFP^+^/Aldh1a2^+^ cells were induced. Interestingly, the expression levels of both GFP and Aldh1a2 in GFP^+^/Aldh1a2^+^ cells were weaker compared to those in GFP^+^ or Aldh1a2^+^ cells (Figure 8F). Expression of Aldh1a2 in immature cells and its progressive decline accompanying the emergence of Scx^+^ cells suggest the regulatory mechanisms of Scx^+^ cell differentiation where cell-autonomous RA signaling acts as an inhibitory regulator. Taking together, RA signaling negatively regulated induction of entheseal chondrogenic differentiation without exogenously added TGF-β2 as well as tenogenic and fibrochondrogenic differentiation with TGF-β2, possibly in a cell-autonomous manner (Figure 8G).

## Discussion

In this study, we successfully generated *ScxGFP* iPSC lines from *ScxGFP* Tg mice and established a novel culture system that recapitulates the dynamic processes of tendon formation observed *in vivo*. Taking advantage of the stepwise differentiation of *ScxGFP* iPS cells into tenocytes, we revealed a tenogenic differentiation trajectory by scRNA-seq analysis.

We screened ligands related to several developmental pathways by using *ScxGFP* iPSCs and found that TGF-β2 was the most potent for the generation of tenocytes. Importantly, induced cells highly expressed Tnmd at both transcript and protein levels. Tnmd is a widely accepted molecular marker of mature tenocytes (Shukunami et al., 2006). Recent studies have also reported the induction of tenogenic cells from *ScxGFP* pluripotent cells by activation of TGF-β signaling using TGF-β1 (Komura et al., 2020; Kaji et al., 2021). In the developing mouse limb, *Tgfb2* was reported to show the highest level of expression compared to those of other *Tgfb* genes (Havis et al., 2014). In addition, *Tgfb2*;*Tgfb3* double mutant embryos showed loss of most tendons and ligaments in the limbs, trunk, tail, and head (Pryce et al., 2009), indicating that TGF-β1 cannot compensate for tenogenic activities of TGF-β2 or 3. In agreement with these findings, our screening revealed that TGF-β2 has a much stronger tenogenic induction potential than TGF-β1. Furthermore, cluster 3 in our scRNA-seq analysis was characterized by high levels of expression of both *Scx* and *Tgfb2*. This suggests the activation of autocrine TGF-β2 signaling, which also operates in tendon progenitors during embryogenesis (Pryce et al., 2009; Havis et al., 2014). Thus, it appears that the protocol established in this study accurately recapitulates the *in vivo* tendon developmental processes and ensures efficient tenocyte induction. As shown in Figure 7F, our protocol led to the induction of 66.23% GFP^+^ cells. Compared with previous reports, the efficiency of generating ScxGFP^+^ cells using our method is greater than that of Komura’s protocol (18%) but lower than that of Kaji’s protocol (90%). Because Kaji et al. used the hedgehog signaling activator SAG in addition to TGF-β1, we also tested the effects of SAG in our protocol. Treatment with TGF-β2/SAG further promoted the induction of GFP^+^ cells, compared to the case for treatment with TGF-β2 alone (Supplementary Figure S7), confirming the stimulatory effects of SAG on TGF-β-driven tenogenic induction.

Sox9 is a key regulatory transcription factor that regulates chondrogenic differentiation. It is also expressed in other mesenchymal cell progenitors of osteoblasts, tenocytes, and ligamentocytes (Akiyama et al., 2005; Sugimoto et al., 2013a; Ideo et al., 2020). During development, Scx^+^/Sox9^+^-lineage cells have the potential to differentiate into tenocytes, ligamentocytes, and entheseal chondrocytes of hyaline cartilage, while tenocytes and chondrocytes away from the prospective enthesis are derived from Scx^+^/Sox9^-^- and Scx^−^/Sox9^+^-lineage cells, respectively (Sugimoto et al., 2013a). Activation of TGF-β signaling, indicated by phosphorylation of Smad3, is observed in the developing patella and deltoid tuberosity derived from the Scx^+^/Sox9^+^-lineage cells (Yoshimoto et al., 2017), suggesting that TGF-β2 induces the conversion of Sox9^+^ cells into Scx^+^/Sox9^+^ cells. The concomitant expansion of Scx^+^ cells upon treatment with TGF-β2 may reflect the fact that Scx^+^/Sox9^+^ cells *in vivo* are only transiently present to generate either Scx^+^ or Sox9^+^ cells (Sugimoto et al., 2013a). However, it is still uncertain whether all of the Scx^+^ cells arose from the Sox9^+^-lineage cells in our culture system. Interestingly, continuous treatment with TGF-β2 suppressed cartilage nodule formation in a dose-dependent manner, while inhibition of TGF-β signaling resulted in enhanced chondrogenic differentiation. The coordinated regulation of cartilage and tendon differentiation by TGF-β2 in chicken mesenchymal micromass cultures has been reported (Lorda-Diez et al., 2009). TGF-β-mediated induction of Scx in paratenon progenitors permits the healing of adult Achilles tendon by suppressing chondrogenesis (Sakabe et al., 2018). Our data suggest that TGF-β2 is involved in the switching of mesodermal progenitors to tenogenic/ligamentogenic lineages rather than the chondrogenic lineage.

The importance of Scx in tendon development has been demonstrated in *Scx-deficient* mice. *Scx* was reported to be required for the formation of long-range tendons and the recruitment of mesenchymal progenitors during elongation of long tendons (Murchison et al., 2007; Huang et al., 2019). It was also reported that *Scx* is required for the expression of tendon-related genes (Murchison et al., 2007; Yoshimoto et al., 2017). Importantly, Tnmd was almost absent in our *Scx-deficient* mice, even in embryonic short tendons and ligaments, with no apparent abnormalities in appearance at this point (Yoshimoto et al., 2017). Conversely, overexpression of *Scx* in the developing hindlimb significantly upregulated *Tnmd* expression exclusively in tenocytes (Shukunami et al., 2006). We also demonstrated that Scx acts as a transcriptional activator of the *Tnmd* gene by binding to E-boxes located in the 5-flanking region of the mouse *Tnmd* locus (Shukunami et al., 2018). Although it is possible that the *Scx* expression is too low to be detected in T9 cells, the scRNA-seq analysis in this study revealed a progressive trajectory where cells are shifting from *Scx* ^+^/*Tnmd* ^−^ to *Scx* ^+^/*Tnmd* ^+^ and finally, to the *Scx* ^−^/*Tnmd* ^+^ state.

In our differentiation system, suppression of RA signaling by BMS493 resulted in increased induction of entheseal chondrocytes, fibrochondrocytes, and tenocytes (Figure 8F). This positive action of BMS493 on chondrocyte induction is consistent with the previous finding that inhibition of RAR activity enhances chondrogenic differentiation (Weston et al., 2002; Shimono et al., 2011). Kaji et al. reported that the activation of RA signaling by ATRA during paraxial mesoderm induction almost entirely eliminated ScxGFP induction by promoting the switch to a neural fate. However, during tenogenic induction by TGF-β1 and the hedgehog signaling activator SAG, they showed that RA signaling promotes tenogenic differentiation (Kaji et al., 2021). In this study, we found that activation of RA signaling significantly suppresses TGF-β-dependent early tenogenic differentiation as evidenced by a decreased number of Scx^+^ cells and that conversely suppression of RA signaling promotes both tenogenic and fibrochondrogenic differentiation (Figures 8A–C). Contradictory actions of RA signaling on tenogenesis reported by this study and Kaji et al. may result from different culture conditions. As reported by Kaji et al., treatment with TGF-β2/SAG further promoted the induction of GFP^+^ cells, compared to the case for treatment with TGF-β2 alone, confirming the stimulatory effects of SAG on TGF-β2-driven tenogenic induction. However, the efficiency of GFP^+^ cell generation after the treatment with TGF-β2/ATRA/SAG remained as low as that observed after treatment with TGF-β2/ATRA, indicating that the inhibitory effects of ATRA are superior to the stimulatory effects of SAG in the tenogenic induction (Supplementary Figure S7). Therefore, contradictory results between our study and Kaji et al.‘s study seem to not be due to the difference in the modulation of hedgehog signaling. In Kaji et al.’s study, paraxial progenitors were induced using CHIR and the BMP inhibitor LDN-193189 (Kaji et al., 2021). In our system, the cells at T0, before the induction of tenogenic differentiation, expressed both paraxial and lateral plate mesoderm markers (Supplementary Figure S6). This may be due to the fact that TGF-β or BMP signaling is not suppressed during mesodermal induction (Xi et al., 2017). Differences in mesodermal cell identity could account for the different responses to RA signaling observed in our study and Kaji et al.’s study. Since the degree of RA inhibition has been reported to be inversely proportional to the level of *Sox9* induction (Weston et al., 2002), under the conditions of RA signaling suppression, we speculate that more Sox9^+^ progenitor cells with the potential to differentiate into chondrocytes and tenocytes could propagate, thus ultimately leading to enhanced differentiation toward these cell fates. Our study further revealed differentiation trajectory where Aldh1a2^+^ RA producing progenitors mature to become Scx^+^ cells through the Aldh1a2^+^/Scx^+^ status, raising the possibility that TGF-β signaling counteracts inhibitory action of cell-autonomous RA signaling on tenogenic differentiation. Although paracrine action of RA signaling was reported to regulate development of the myotendinous junction in extraocular region (Comai et al., 2020), regulation of tenogenesis by cell-autonomous action of RA has not been reported. Further studies will be required for comprehensive understanding of roles for RA signaling in the regulation of tendon formation *in vivo*.

In conclusion, we developed a tenogenic induction method using the originally established *ScxGFP* iPSC lines. scRNA-seq analysis revealed a tenogenic differentiation trajectory, which accurately reflected *in vivo* tenocyte differentiation. The scRNA-seq dataset highlighted inhibitory role of RA signaling in the regulation of tenogenesis. Our tenogenic induction method and scRNA-seq data will provide valuable information for a better understanding of tendon and ligament biology.

In this report, we were not able to evaluate the contribution of the generated tendon cells to tissue formation. For the further improvement of the system, it is necessary to further examine the tendon cells generated herein by performing *ex vivo* tendon formation and transplantation after tendon injury in future studies.

## Data Availability

The datasets presented in this study can be found in online repositories. The data presented in the study are deposited in the GEO, accession number GSE168451. All datasets generated for this study are included in the article/Supplementary Material.
