# Supplementary material for: Tenogenic Induction From Induced Pluripotent Stem Cells Unveils the Trajectory Towards Tenocyte Differentiation
Source: Front Cell Dev Biol. 2022 Mar 9;10:780038. doi: 10.3389/fcell.2022.780038 (PMC8965463; doi:10.3389/fcell.2022.780038)
Supplement: Supplementary file 1 [file DataSheet1.PDF]

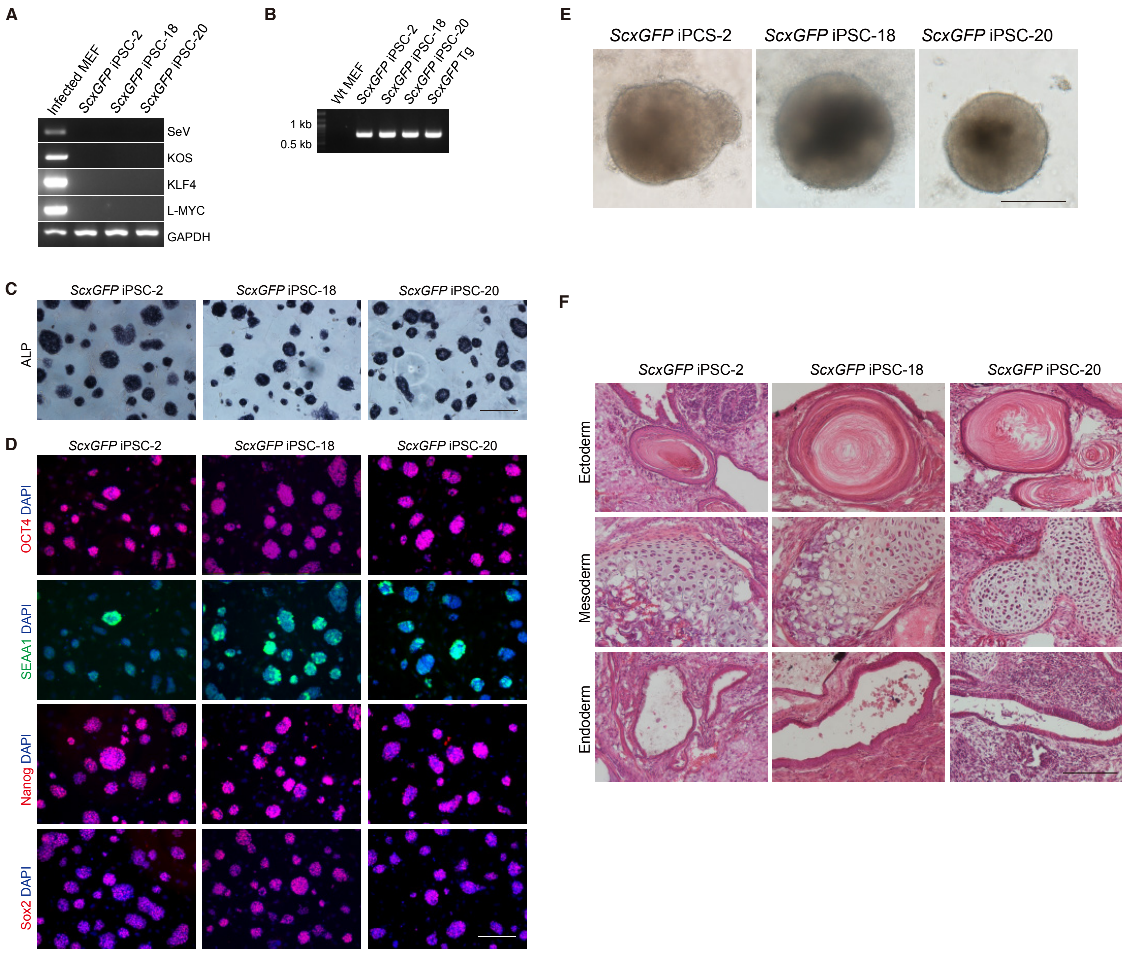

Supplementary Figure 1. Characterization of established *ScxGFP* iPSCs. (A) RT-PCR using primers for detection of Sendai virus vectors (SeV, KOS, KLF4, L-MYC) in infected MEFs and established *ScxGFP* iPSCs. (B) Genomic PCR for detection of the *ScxGFP* transgene in wild type MEFs, *ScxGFP* iPSCs, and tail tip of *ScxGFP* Tg mice. (C) Alkaline phosphatase (ALP) activity in *ScxGFP* iPSCs. A high level of ALP activity is detected in established iPSCs. (D) Immunofluorescent staining for OCT4, SEAA Nanog, and Sox2 in established *ScxGFP* iPSCs. (E) Embryoid bodies formation from *ScxGFP* iPSC-2, 18, and 20. (F) HE-staining of teratomas derived from *ScxGFP* iPSC-2, 18, and 20. Regions including ectoderm (upper row), mesoderm (middle row), and endoderm (lower row) are presented. Scale bar, 200 µm (C–F).

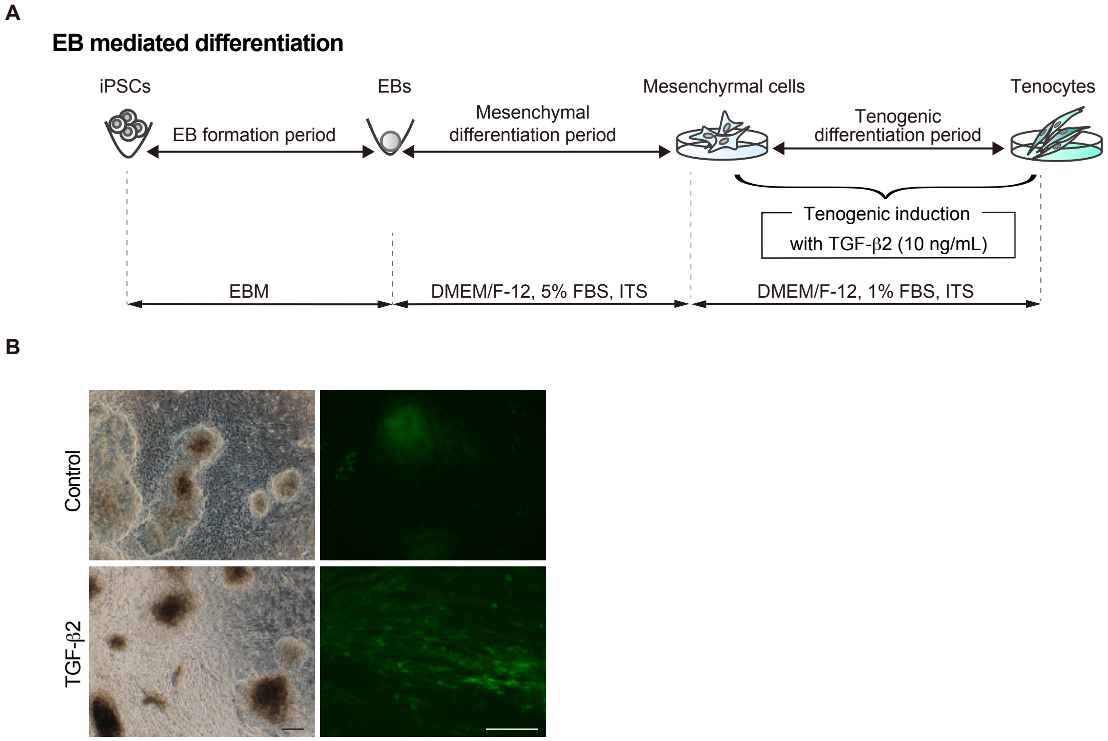

Supplementary Figure 2. Embryoid body (EB) mediated *in vitro* differentiation. (A) Schema of EB formation-based *in vitro* differentiation. Mesenchymal cells were obtained by passages of outgrown cells from EBs. Induced mesenchymal cells were subject to tenogenic induction by TGF-β2. (B) Phase (left column) and fluorescent (right column) images of induced cells by TGF-β2. Scale bar, 200 μm.

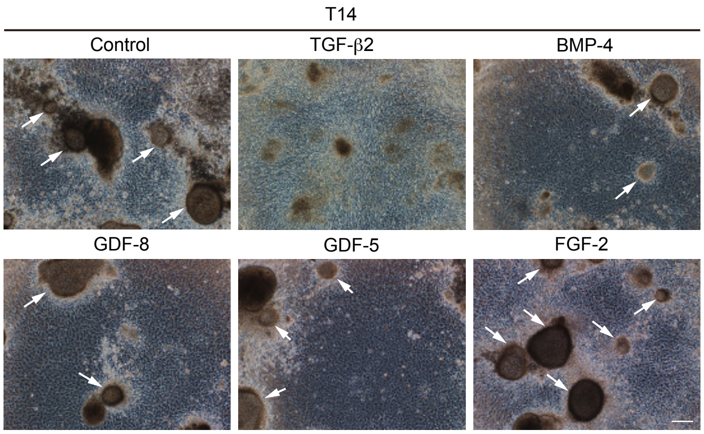

Supplementary Figure 3. Effects of various growth factors on chondrogenic differentiation in *ScxGFP* iPSC cells. Phase contrast images of control cells and cells treated with various growth factors for 14 days (T14). Arrows indicate the cartilaginous nodules. Scale bar, 200 μm.

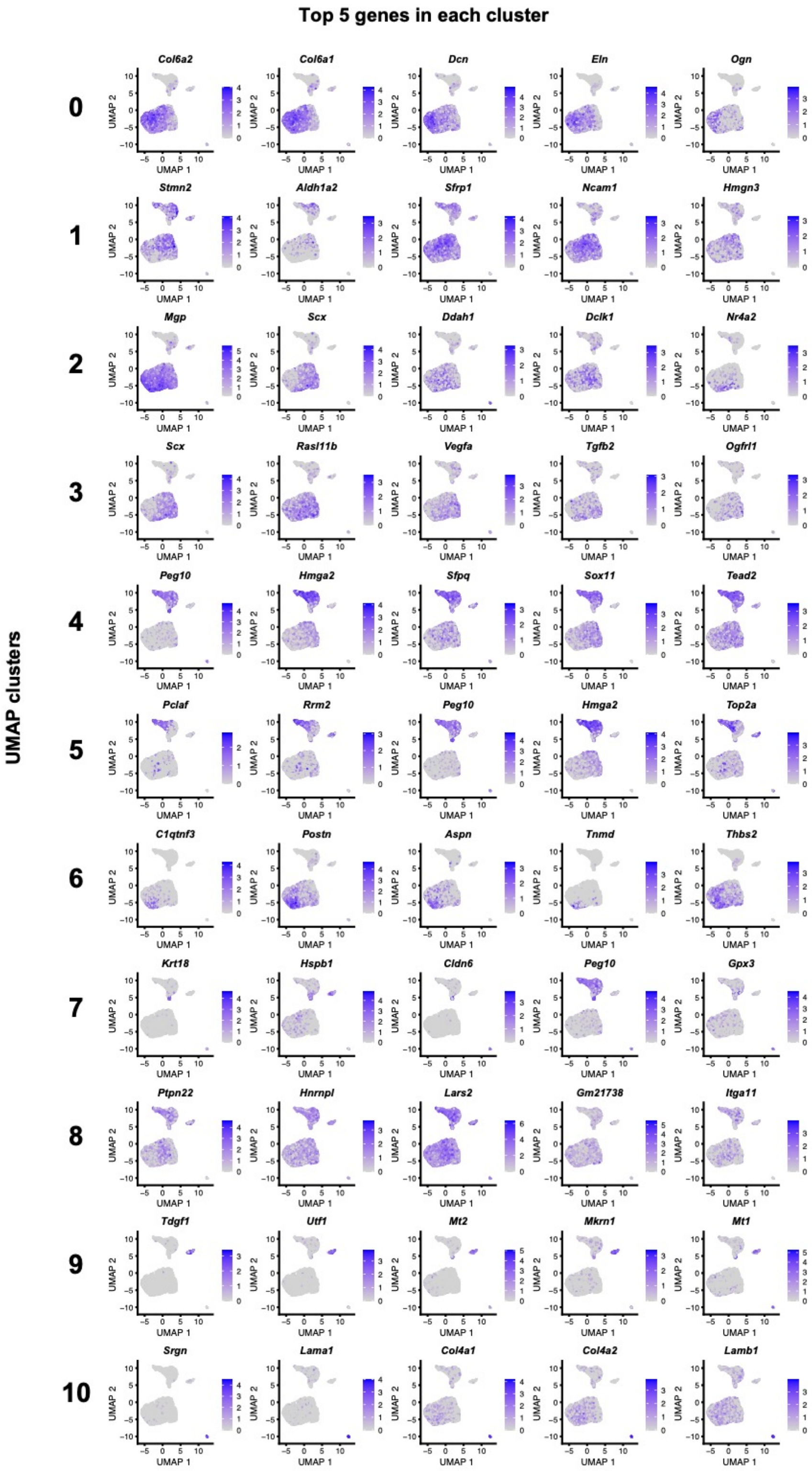

Supplementary Figure 4. Top five representative genes in UMAP clusters. UMAP plots showing the expression levels of the top five representative genes in each UMAP cluster. The expression levels are expressed by blue to gray gradient color.

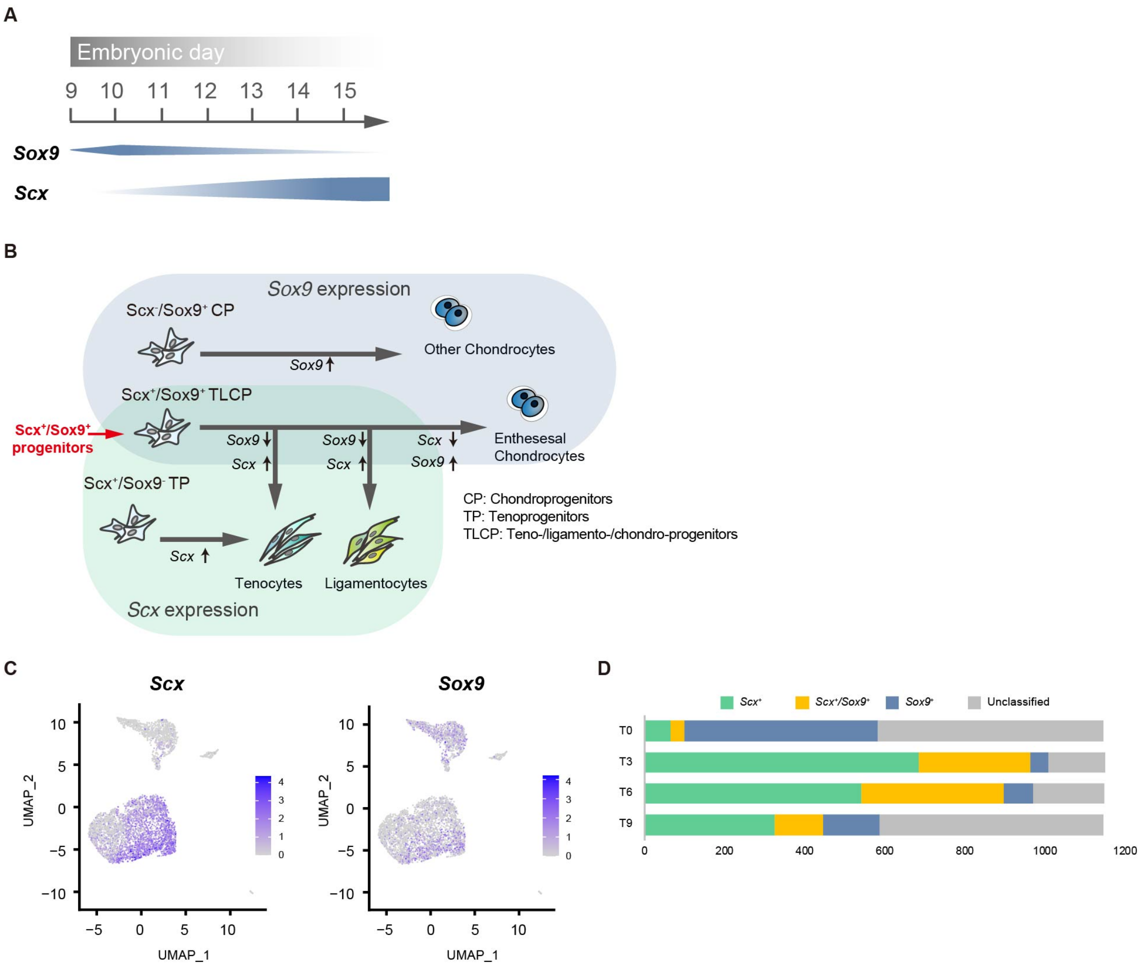

Supplementary Figure 5. Scx/Sox9 expression status. (A) Schematic illustration of Scx and Sox9 expression during embryonic development. (B) Schematic illustration showing the tenogenic, ligamentogenic, and chondrogenic cell lineages along the Scx/Sox9 axis. (C) UMAP plots showing the expression levels of *Scx* and *Sox9*. Expression levels are expressed by blue to gray gradient color. (D) Number of *Scx*$^{+}$ (green), *Scx*$^{+}$/*Sox9*$^{+}$ (yellow), and *Sox9*$^{+}$ (blue) cells in each time point is shown.

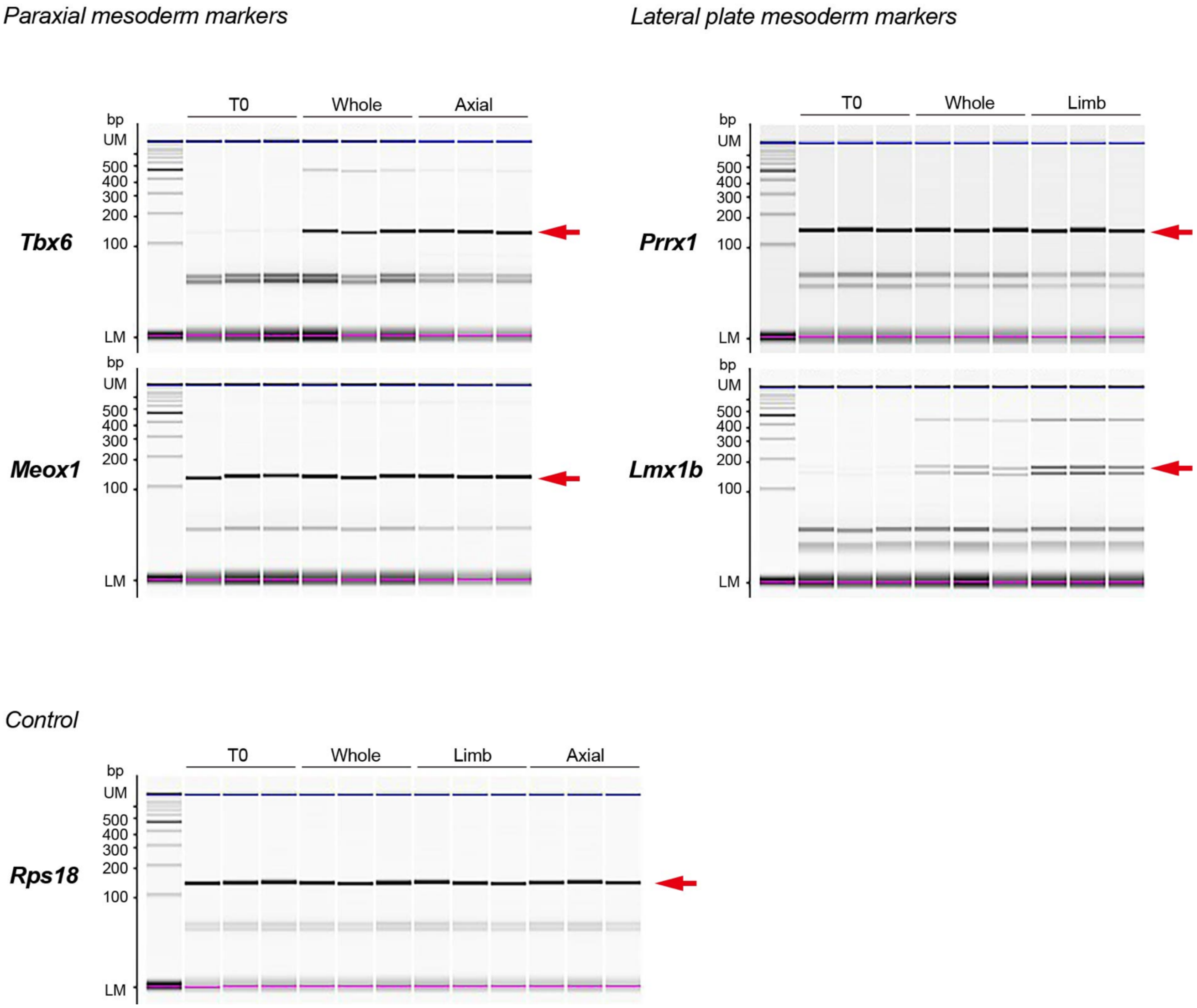

Supplementary Figure 6. Expression of paraxial and lateral plate mesoderm markers in T0 cells. Expression of marker genes for paraxial (*Tbx6* and *Meox1*) and lateral plate (*Prrx1* and *Lmx1b*) mesoderm in T0 cells were analyzed by RT-PCR. Whole bodies (Whole), trunk tissues (Axial), and limbs (Limb) of murine embryos at E11.5 were used as positive control. Expression of *Rps18* is detected in all samples. Red arrowheads indicate positive bands. A lane for size marker was presented at left side. Upper and lower markers (UM and LM) were used to adjust each lane.

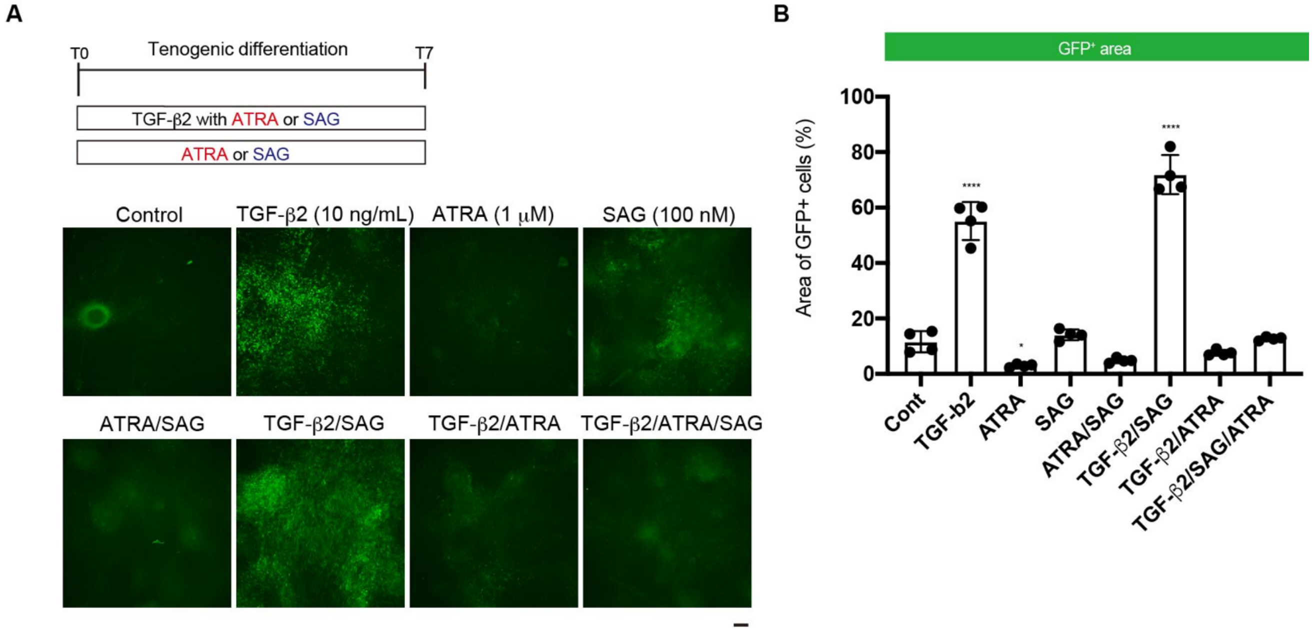

Supplementary Figure 7. Retinoic acid negatively regulated induction of Scx$^+$ cells even in the presence of SAG, an activator of hedgehog signaling. (A, B) Effects of retinoic acid signaling on tenogenic induction in the presence of hedgehog signaling were investigated by using all-trans retinoic acid (ATRA) and SAG with TGF-β2. Immunofluorescent staining for GFP at T7 (A) and percentage of GFP$^+$ areas (B) in each condition were presented. The data represent the mean ± SD (n = 4). One-way ANOVA followed by Dunnett's multiple comparison test. *$P < 0.05$, ****$P < 0.0001$.

Supplementary Table 1. List of primer sets for RT-qPCR, RT-PCR, and genotyping PCR in this study

Target	Forward Sequence (5’–3’)	Reverse Sequence (5’–3’)	Experiment
EGFP	AGATCCGCCACAACATCGAG	TCTCGTTGGGGTCTTTGCTC	RT-qPCR
Scx	GCGAGAACACCCAGCCCAAACAG	CACGGTCTTTGCTCAACTTTCTCTG	RT-qPCR
Tnmd	TGCTGGATGAGAGAGGTTACTG	GTTGCAAGGCATGATGACAC	RT-qPCR
Mkx	ATTATGTGTCACCGCCCAAG	AGCTAAAAGACCCCGAATGG	RT-qPCR
Col1a2	CCAGCGAAGAACTCATACAGC	GGACACCCCTTCTACGTTGT	RT-qPCR
Rps18	AAGTTCCAGCACATTTTGCGAGTA	TTGGTGAGGTCGATGTCTGCTTTC	RT-qPCR<br>RT-PCR
Nanog	AGGGTCTGCTACTGAGATGCTCTG	CAACCACTGGTTTTTCTGCCACCG	RT-qPCR
Sox2	CACAACTCGGAGATCAGCAA	CTCCGGGAAGCGTGTACTTA	RT-qPCR
T	GAACCTCGGATTCACATCGT	TTCTTTGGCATCAAGGAAGG	RT-qPCR
Mixl1	CGACAGACCATGTACCCAGA	AGGGCAATGGAGGAAAACTC	RT-qPCR
Sox9	CATCAAGACGGAGCAGCTGAG	ATGGTCAGCGTAGTCGTATTG	RT-qPCR
Egr1	CAGCGCCTTCAATCCTCAAG	GAGCGATGTCAGAAAAGGACTC	RT-qPCR
Sev	GGATCACTAGGTGATATCGAGC	ACCAGACAAGAGTTTAAGAGATATGTATC	RT-PCR
KOS	ATGCACCGCTACGACGTGAGCGC	ACCTTGACAATCCTGATGTGG	RT-PCR
KLF4	ACAAGAGAAAAAACATGTATGG	CGCGCTGGCAGGGCCGCTGCTCGAC	RT-PCR
L-MYC	AGTCTCTGGGTATTCGGAAG	CTGGATAGAGTATGTCAGAAGGGTTTTG	RT-PCR
Acan	CAGAACCTTCGCTCCAATGAC	CAGGGTGTAGCGTGTGGAAA	RT-qPCR
Col2a1	ATCTTGCCGCATCTGTGTGT	GGCCCTAATTTTCCACTGGC	RT-qPCR
Col11a1	ACGGAGGAAGCACAAATCAG	CATTGTAGAGGGACAAGAGGAAAG	RT-qPCR
Gapdh	ACCACAGTCCATGCCATCAC	TCCACCACCCTGTTGCTGTA	RT-PCR

Tbx6	TCTACACCCTGCCGCTTTTC	TCCAGGCTGTAGGTCCAGAAA	RT-PCR
Meox1	CCCACCACAACTACCTGACC	CCCTTCACACGTTTCCACTT	RT-PCR
Prrx1	CCAGAGTGCAGGTGTGGTTT	GGACGAGGTACGATGGGTTG	RT-PCR
Lmx1b	GCAGAGCCCCTACGGAAGTA	GAAGAGCCGAGGAAGCAGTC	RT-PCR
ScxGFP	ATGGTGAGCAAGGGCGAGGAGCTGTTC	GGAAGATCTGTTACTTGTACAGCTCGTCC	Genomic PCR

Supplementary Table 2. Mapping summary

												per used cell
plate(384cells)	total read	trimmed read	Unmapped	Multi-mapped	NoFeatures	Ambiguity	Assigned	umi count	umi duplicate	used cell number*	umi count per cell (median)
T0-1	35,090,556	1,064,969	9,380,883	8,711,967	3,806,992	760,307	11,365,438	4,950,455	6,414,983	381	12,987
T0-2	25,135,920	811,811	7,030,473	6,554,974	2,640,673	543,708	7,554,281	4,591,413	2,962,868	383	11,986
T0-3	23,849,154	790,103	6,868,492	6,452,916	2,406,160	550,556	6,780,927	3,928,452	2,852,475	382	10,281
T3-1	18,619,607	218,623	3,524,924	5,336,733	1,153,115	612,198	7,774,014	2,203,161	5,570,853	384	5,737
T3-2	23,850,370	615,408	6,134,415	5,353,243	3,200,422	512,577	8,034,305	3,058,851	4,975,454	383	7,987
T3-3	21,135,238	513,904	4,707,247	5,878,720	1,668,880	628,771	7,737,716	3,340,158	4,397,558	384	8,698
T6-1	26,672,947	867,259	7,743,197	6,364,117	3,255,712	525,696	7,916,966	4,299,391	3,617,575	382	11,249
T6-2	23,772,438	572,995	5,443,909	7,200,840	1,528,100	763,582	8,263,012	3,530,641	4,732,371	384	9,194
T6-3	25,961,677	841,620	7,596,465	6,313,725	3,182,924	540,566	7,486,377	4,614,190	2,872,187	383	12,044
T9-1	19,840,602	268,524	3,986,534	6,325,885	1,370,361	694,537	7,194,761	4,144,731	3,050,030	382	10,849
T9-2	21,611,631	555,722	4,905,698	6,850,468	1,505,225	752,757	7,041,761	3,476,112	3,565,649	381	9,114
T9-3	18,105,738	416,540	4,258,534	4,262,067	2,156,494	399,294	6,612,809	3,293,163	3,319,646	383	8,596

* cells expressed > 200 genes

Supplementary Table 3. Mean Ct values for each target gene

Mean Ct values for each target gene in Figure 3C						
**Gene**	**Control**	**TGF-β2**	**BMP-4**	**GDF-8**	**GDF-5**	**FGF-4**
**EGFP**	27.5	20.3	25.7	27.1	26.4	27.1
Rps18	18.6	18.3	18.5	18.4	18.7	18.4
**Scx**	24.7	20.9	24.6	24.5	24.5	24.4
Rps18	18.8	18.5	18.8	18.8	18.8	18.5
**Tnmd**	25.7	19.4	26.6	22.7	25.8	24.5
Rps18	18.8	18.5	18.8	18.8	18.8	18.5

Mean Ct values for each target gene in Figure 5C					
**Gene**	**iPSC+MEF**	**M4**	**T-1**	**T7**	**T14**
**Nanog**	19.4	21.3	26.2	25.5	25.8
Rps18	17.9	18.1	18.6	18.7	18.5
**Sox2**	25.3	28.2	31.8	31.4	31.0
Rps18	17.9	18.1	18.6	18.7	18.5
**T**	25.5	24.0	27.1	26.9	29.7
Rps18	18.2	18.3	18.8	18.9	18.8
**Mixl1**	29.1	27.7	30.7	31.4	30.8
Rps18	18.2	18.3	18.8	18.9	18.8
**Sox9**	28.9	27.0	23.4	22.9	24.9
Rps18	18.0	18.2	18.7	18.8	18.7
**Scx**	28.8	27.3	28.0	21.8	23.5
Rps18	18.0	18.2	18.7	18.8	18.7
**Mkx**	27.6	27.4	26.3	22.3	23.0
Rps18	18.1	18.3	18.7	18.9	18.7
**Tnmd**	27.7	32.2	33.6	17.4	18.4
Rps18	18.0	18.2	18.7	18.8	18.7
**Egr1**	29.3	26.0	24.7	21.9	20.2
Rps18	18.1	18.3	18.7	18.9	18.7
**Col1a2**	19.4	24.1	23.0	16.9	16.6
Rps18	18.0	18.2	18.7	18.8	18.7
